# Quantifying Epistemic Relevance

**DOI:** 10.1162/OPMI.a.332

**Published:** 2026-04-14

**Authors:** Alex Warstadt, Omar Agha, Michael Franke

**Affiliations:** University of California San Diego, La Jolla, CA, USA; University of Maryland, College Park, MD, USA; University of Tübingen, Tübingen, Germany

**Keywords:** relevance, discourse, experimental pragmatics, information theory, Bayesian epistemology

## Abstract

In ordinary information-seeking dialogue, the relevance of an assertion seems to depend on how much progress it makes toward resolving the question under discussion (QUD). This kind of relevance—epistemic relevance—has been quantified in more than one way in prior work. However, there has been little investigation into which measures of epistemic relevance best model human judgments. We present the most comprehensive (to date) experimental evaluation of different candidate notions of epistemic relevance applied to question-answer pairs. We find that a measure based on the Bayes factor, which quantifies the amount of evidence provided by the answer, is the best predictor of relevance ratings for responses to polar questions, outperforming other information-theoretic measures based on Kullback-Leibler divergence and change in entropy. We compare models that use first-order beliefs (point estimates) and models that use second-order beliefs (probability density functions over probabilities). Our findings suggest that both first-order and second-order beliefs play a role in predicting introspective judgments. However, intuitions about relevance are not purely epistemic, meaning factors other than belief change are needed to fully characterize what it means to be relevant.

## INTRODUCTION

Prominent theories of pragmatics operate under the assumption that human conversational behavior is, to some extent, rational (Benz et al., 2006; Grice, 1975) or optimal (Blutner & Zeevat, 2004; Sperber & Wilson, 1995). By common definition, an agent’s behavior is rational or optimal if it is maximally conducive to reaching the agent’s goals, to the extent possible. Formally, this is often framed as maximizing a measure of (expected) utility, e.g., in rational choice theory (e.g., Raiffa & Schlaifer, 1961; Savage, 1951). Yet it is not exactly clear what notion(s) of utility are most useful for explaining human communicative behavior. Linguistic analysis has come a long way by focusing on cooperative, information-seeking dialogue, and equating usefulness of a conversational move with its informativity or relevance (Grice, 1975; Sperber & Wilson, 1995). But there is an abundance of formal notions quantifying, in various forms, information flow and relevance, drawing on information theory, Bayesian epistemology, decision making or statistical inference. This paper therefore addresses the empirical question of which quantitative measures of information exchange best align with participants’ intuitive judgments of usefulness.

We focus on the special case of quantitative measures of *epistemic relevance*, as we call it here, in answers to polar questions, like in example (1). An answer like (1-a) is a candidate for a communicative act that is deemed irrelevant since it provides neither epistemically nor practically useful information. Some intuitively relevant answers, like (1-b), do not answer the concrete question at all, thus provide no information on the actual issue, but may be deemed *practically relevant* if they address the reason why the question was asked in the first place (cf. Hawkins & Goodman, 2017; Stevens et al., 2016; Tsvilodub et al., 2023).^1^ But there is also a large range of answers which do, in principle, address the issue raised by the polar question, and so should count as epistemically relevant, but only make partial progress toward resolving the issue. Within this class of answers, there seem to be degrees of epistemic relevance, hinging on, among other things, the strength of the information provided. For example, answer (1-c) only provides little information, and may therefore be deemed less relevant than more informative answers like in (1-d) or (1-e).

(1)  Q: Will the wedding be outside?
    a. I am going to wear high heels no matter the weather.        [irrelevant]
    b. I will be close by with an umbrella.           [practically relevant]
    c. It’s cloudy.                    [low epistemic relevance]
    d. Forecast said rain was likely.          [medium epistemic relevance]
    e. It already started to rain.             [high epistemic relevance]

**Figure 1.**
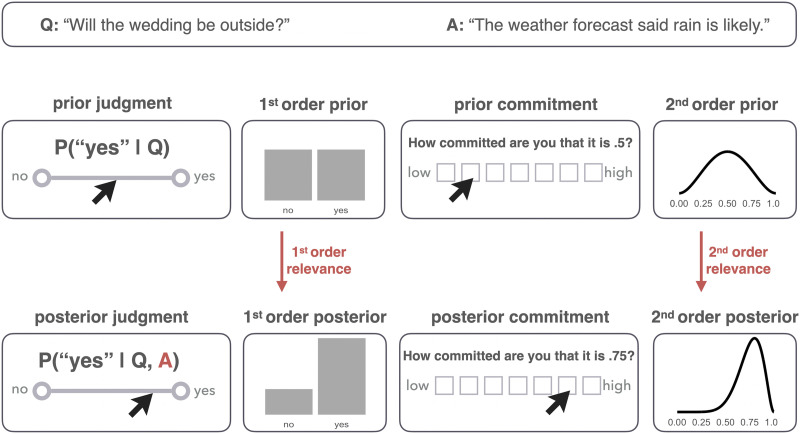
Overview of experimental design and measures of relevance. In each trial, participants indicated their prior belief about ‘yes’ answer to a polar question, followed by a commitment rating. After reading the answer, they again gave a probability judgment and a commitment rating again. Probability ratings indicate participants’ first-order beliefs (a probability distribution over answers ‘no’ and ‘yes’). Additional commitment ratings allow estimation of a second-order belief as well (a probability distribution over all probabilities of ‘yes’ answers). Relevance of information is assessed as a measure of difference between the prior and posterior beliefs.

Figure 1 provides a schematic overview of the experimental and modeling approach presented here. For one, the paper introduces novel experimental methods for gathering empirical data relevant to studying human intuitive judgments of relevance. Concretely, we build on previous like-minded approaches (Agha & Warstadt, 2020; Warstadt & Agha, 2022), to gather experimental data on subjects’ prior and posterior beliefs about a binary issue, i.e., their estimate of the probability of a ‘yes’ answer before and after hearing the answer to a polar question. By also collecting participants’ ratings of commitment to their probability judgments, we gain information about their second-order uncertainty as well, i.e., their beliefs about how likely different probability judgments for a ‘yes’ answer might be (Fagin & Halpern, 1994; Herbstritt & Franke, 2019; Moss, 2015). The paper’s main technical contribution is a stringent comparison of different quantitative notions of relevance, taken from information theory, Bayesian epistemology and statistics, all of which define epistemic relevance as a measure of belief change from the prior to the posterior. We compare salient candidate notions from the literature based on how well they explain human judgments of relevance of information in answers to polar questions similar to examples (1-c) through (1-e).

Our results identify the Bayes factor utility as the measure that best predicts human relevance judgments. The Bayes factor is commonly used in Bayesian statistics to quantify how much a new piece of information shifts probability in favor of a particular explanation. However, Bayes factors based on both first-order and second-order belief states together predict relevance better than either on its own, suggesting that no single measure fully explains relevance. In addition to a rigorous quantitative comparison of measures, we perform a qualitative analysis of results which reveals that, in some cases, participants attributed a substantial degree of relevance to an answer while at the same time indicating that they themselves did not change their beliefs in response to that answer. Upon inspection of examples, we identify a number of subtle factors which might influence relevance judgments on top of the epistemic relevance based on the listener’s own beliefs, e.g., by judging relevance for a generic listener other than oneself. We also conduct a “relevance-only” followup experiment in which participants give relevance judgments without being prompted to directly consider their probabilistic judgments or changes in belief state. While this change in methodology lowers the overall fit of measures of epistemic relevance to introspective judgments, the relative fit of different competing measures remains the same.

The paper is structured as follows. Utility, Relevance, and Information section surveys key ideas from the previous relevant literature on how to quantify usefulness of information. First-Order and Higher-Order Beliefs section expands on the distinction between first- and second-order beliefs, and motivates why the latter are important for considerations of epistemic relevance. Candidate Measures of Epistemic Relevance section introduces several candidate notions for quantifying relevance. Experiment 1: Individuals’ Beliefs Predict Relevance Judgments section introduces our main experimental method (Experiment 1) and reports the results. Experiment 2: Introspection on Belief States Affects Relevance Judgments section presents the methods and findings of the “relevance-only” followup study (Experiment 2). General Discussion and Future Work and Conclusion sections discuss the results and qualitative analysis, reflect on limitations and future work, and conclude.

## UTILITY, RELEVANCE, AND INFORMATION

Human actors have complex goals, relating to the current conversation, but also involving many other non-linguistic aspects of their lives. Yet, for the sake of concrete modeling, some prior works consider communicative behavior from the perspective of agents maximizing a single measure of overall utility. This enables investigating abstract questions about the nature of communication, such as investigating the conditions under which conventional codes can develop if agents are either fully cooperative (e.g., Lewis, 1969; Nowak & Krakauer, 1999; Skyrms, 2010; Wärneryd, 1993) or to some extent uncooperative (e.g., Crawford & Sobel, 1982; Spence, 1973; Wagner, 2015). However, as these high-level approaches focus on population models or make strong assumptions about agents’ rationality to explain abstract choice patterns in recurring situation types, they are less explanatory for how human agents with rich routines of contextual decision making evaluate their choice options in any given concrete conversational situation.

Explanations of conversational behavior and interpretation have been strongly influenced by the seminal work of Grice (1975), suggesting that, by and large, a speaker’s communicative behavior can be described as following a set of principles, the so-called **Maxims of Conversation**, according to which speakers usually act in such a way that they adhere to standards of truth and justifiability through evidence, express themselves clearly and seek to provide, cooperatively, just the right amount of relevant information that is needed at the current point of the conversation. Yet Grice’s work left largely unexplored how to specify notions of informativity and relevance further.

Formal work in linguistics which followed a logical tradition (e.g., Gamut, 1990) took up Gricean ideas by identifying informativity of an expression as **logical strength**. An expression *φ* is more informative than another *ψ* if *φ* logically entails *ψ*. A problem with this approach is that it only induces a partial ordering on expressions, even where we have clear intuitions about differences in informativity. For example, intuitively the sentence in (2-a) provides more information (e.g., about the results of an exam) than (2-b). Yet these sentences are logically independent, thus not comparable in terms of a naïve notion of informativity as logical strength.

(2)  a. Most of the students got an A.
    b. John got an A.

A popular and productive approach to formalizing relevance in Grice-inspired formal linguistics uses the concept of a **Question Under Discussion (QUD)** (Ginzburg, 1996; Roberts, 2012; Van Kuppevelt, 1995). In this view, at any point in a conversation, there is a question (often assumed to be the top of a stack of questions) representing the current topic of conversation: the current informational goal of the listener is to resolve the QUD. A conversational move is predicted to be relevant if and only if it contributes to this goal by eliminating a possible answer to the QUD. A closely related contribution is the idea that conversational agents keep track of and measure informativity relative to the current belief states of the conversational agents. Stalnaker (1978) expressed this intuition in a possible worlds semantics as the **context set**, the set of worlds which are still live possibilities given the established common knowledge in the discourse.

### Issues in QUD Theory

The logical-semantic approach to formalizing pragmatic utility has several seemingly insurmountable limitations. First, the notion of QUD relevance assumes a fixed QUD known to all participants. In actual discourses, the QUD is often implicit—it may not correspond to any overt question. Discourse participants are often uncertain about the QUD, and may need to infer a QUD in order to maintain cooperativity. In this paper, we consider only cases where there is an overt question, and we adopt the simplifying assumption that asking an overt question fixes the QUD. In some cases, this assumption can be harmless, but it is in general not guaranteed to be true. However, the measures of epistemic relevance we discuss also apply in cases where there is an implicit, unambiguous QUD. For work (particularly in the RSA literature) that explicitly models uncertainty about the QUD or other aspects of the conversational context, see Kao et al. (2014), Qing et al. (2016), and Warstadt (2022).

Second, the standard QUD theory of relevance (Roberts, 2012) breaks down in the case of intuitively relevant, yet non-resolving answers to polar questions as in (3). The only responses that eliminate a possible answer to a polar QUD are exhaustive answers that entail the ‘yes’ answer or the ‘no’ answer, leading to the incorrect prediction that the response in (3) should be irrelevant (Agha & Warstadt, 2020).

(3)  A: Is the picnic canceled?
    B: If it rains.

Third, these views make the simplifying assumption that relevance and possibility are binary concepts. To the contrary, there is good evidence that introspective relevance judgments are gradient, and related in some way to the likelihood of the possible answers (Warstadt & Agha, 2022), as suggested by (1).

**Relevance theory** (Sperber & Wilson, 1995) places the notion of relevance at the center of a cognitive-evolutionary explanation of language interpretation. According to relevance theory, human cognition is oriented towards making sense of our environment. For the special case of language, this entails that interpreters will automatically seek to find relevant implications of a speaker’s utterance. A characterization of relevance in this framework is:

“[T]he greater the positive cognitive effects achieved by processing an input, the greater the relevance, […] the greater the processing effort expended, the lower the relevance of the input to the individual at that time.” (Wilson & Sperber, 2004, 610)

A problem with this definition of relevance is that it does not entail a clear objective, and ideally numerical, measure. While it might be possible to give a relevance-theoretic explanation of the *order* of intuitive relevance between sentences (1-c), (1-d) and (1-e), it is not possible to derive from relevance theory predictions about the degree to which (1-e) is more relevant than (1-c), as compared to (1-d), for example.^2^

### Quantitative Measures for Information Value

Quantitative measures for the practical value of information have been suggested in **statistical decision theory** (Jeffrey, 1965; Raiffa & Schlaifer, 1961) and applied to the analysis of language use, for example, in the pioneering work of Parikh (1991). A potential problem with decision-theoretic notions of relevance is that they require the specification of actions a decision maker can perform and numerical utilities associated with the outcomes of these actions. Agents have clear intuitions about the purely informational, epistemic relevance of answers like (1-c), (1-d) and (1-e), even when their possible actions and the utilities of those actions are unspecified. Indeed, some common decision-theoretic notions of relevance of information for practical purposes reduce to standard information-theoretic measures (van Rooy, 2004). For our present purposes, we therefore ignore decision-theoretic notions of practical relevance in favor of information-theoretic notions to quantify epistemic relevance (see Hawkins et al., 2025; Sumers et al., 2024, for recent related work exploring decision makers being informed by a mixture of epistemic and practical relevance).

Notions from **information theory** (Cover & Thomas, 1991; Shannon, 1948) have been used in different kinds of models of language use and interpretation. For example, the Rational Speech Acts (RSA) framework (Frank & Goodman, 2012) is an attempt to implement Gricean pragmatics using the tools of Bayesian cognitive modeling. One key ingredient of the RSA formulation is a utility function that influences how a rational speaker samples an utterance. A prototypical example is given in (4):

(4)  *P*_*S*_(*u* | *w*) ∝ *exp*(*α* · *Util*_*S*_(*u*; *w*))
    *Util*_*S*_(*u*; *w*) = log *P*_*L*_(*w* | *u*) − *Cost*(*u*)

The speaker’s probability, *P*_*S*_(*u* | *w*), of choosing utterance *u* in world state *w* is defined via soft-maximizing (Franke & Degen, 2023) a utility function, which in turn contains a term for the cost of the utterances, which we will neglect here, and the log probability that a listener *L* would assign to the actual world *w* conditioned on the speaker’s utterance *u*. This amounts to the claim that part of the speaker’s linguistic goal in making an utterance is to minimize information-theoretic *surprisal*.

The formulation of the speaker’s utility function in (4) is derivable from a more general assumption, namely that the measure that guides the speaker’s choice of utterance is to minimize the (information-theoretic) distance between their own beliefs about the true world state and that of the listener after the listener has heard a particular utterance (Goodman & Stuhlmüller, 2013; Scontras et al., 2017). If distance between beliefs is defined in terms of the *Kullback-Leibler divergence*, the formulation in (4) is the special case in which the speaker is completely certain that the true world state is *w*. Other likeminded work, e.g., on strategic choice of questioning for efficient information foraging (Rothe et al., 2016, 2018, 2019), has employed a conceptually related, but numerically different notion of usefulness of information, namely *entropy reduction*. Both Kullback-Leibler divergence and entropy reduction are therefore clearly candidate notions for quantifying degrees of epistemic relevance in the context of communication, which we will investigate subsequently.

Next to information-theoretic measures, there are also **Bayesian approaches** to consider. If we consider a polar question as inducing a binary issue, with two competing, mutually exclusive hypotheses ‘yes’ and ‘no’ , Bayesian notions of evidence of information capture how much a given answer changes a recipient’s beliefs in favor of either answer. Widely used Bayesian measures of (observational) evidence use the likelihood ratio, also called the Bayes factor (Good, 1950; Jeffreys, 1961; Kass & Raftery, 1995), quantifying how much more likely the proposition *u* would be true if the answer to the question was ‘yes’ than if it was ‘no’:

(5)  Likelihood ratio: *P*(*u* | ‘yes’) / *P*(*u* | ‘no’)

Applications of this Bayesian measure of evidential strength to language analysis have been championed by Merin (1999), and subsequently used elsewhere (e.g., Cummins & Franke, 2021; van Rooij, 2004; Winterstein, 2012). We will therefore also consider a likelihood-ratio based Bayesian measure as a candidate notion for quantifying epistemic relevance.

In sum, there are many different approaches to characterizing informativity and relevance which have been used in the literature to explain aspects of language use and interpretation. Not all of these lend themselves to a quantitative analysis of human judgments. Of those that do, we consider in particular the information-theoretic measures Kullback-Leibler divergence and entropy reduction, as well as the Bayesian likelihood ratio (or Bayes factor) as useful contenders made salient by their previous use in the literature.

## FIRST-ORDER AND HIGHER-ORDER BELIEFS

Most measures to estimate informational utility depend on the degree to which new information changes an agent’s probability distribution over answers to the QUD. However, experiments by Warstadt and Agha (2022) uncovered numerous instances where relevant information (as judged by native speakers) leads to little or no change in probabilities from prior to posterior. They attribute this perplexing finding to the simplifying assumption that belief states are represented as a single probability distribution over the answers to the QUD. In fact, producing such probability judgments is an unnatural and noisy process for humans. If you ask someone who has just submitted a college application what their chances are of being admitted, their answer may be different from day-to-day, even if there has been no change in information. This suggests that our point probability judgments are actually sampled from a **second-order distribution**, i.e., a probability density function over probabilities (Fagin & Halpern, 1994; Herbstritt & Franke, 2019; Moss, 2015).

### Example: Higher-Order Beliefs

Consider a concrete, albeit stylized example. The (binary) question at issue is whether the next apple drawn from Smith’s barrel (with replacement) will be red or green. Jones has observed three apples drawn from Smith’s barrel already: two red, one green. Based on these observations, Jones’ best guess about the probability of the next apple being red is a single number *p*.^3^ This is a first-order belief, a belief that assigns a single probability to both outcomes: *p* for red, (1 − *p*) for green (see also Figure 1).

No matter what *p* is, Jones is not completely certain that this is the true probability of the next apple being red. After all, Jones only had very little observational evidence, which is compatible with other estimates as well. In other words, Jones may also have higher-order uncertainty, i.e., uncertainty about their uncertainty: They are not only uncertain about the outcome of the next draw (captured in probability *p*), but they are also uncertain about that uncertainty (whether it’s *p* or *p*′). Formally, we may model this as a second-order probability distribution, namely a probability distribution over probability distributions (see Figure 1).

Now, imagine Jones peers into the barrel and observes the 20 apples on top, or Miller tells Jones that they have inventoried all the apples and shared the true proportion of red apples. Furthermore, suppose that by some coincidence, this new information happens to confirm Jones’s original point estimate of *p*. There is a sense in which this information is and is not relevant. On the one hand, Jones’ first-order belief does not change at all, so the new information had no epistemic relevance for Jones: they thought that already. But on the other hand, viewing a larger sample from the barrel has reduced Jones’ higher-order uncertainty somewhat, and Miller’s information has reduced it entirely.

To conclude, this stylized example aims to demonstrate how utterances that are entirely uninformative at a first-order level of beliefs can affect higher-order uncertainty. There is a sense in which these utterances are intuitively relevant, and so we should also include higher-order epistemic relevance, at least to the second-order, in our investigation.

### Representing Second-Order Belief States

In general, a second-order belief state can be any probability density function over first-order belief states. To simplify matters, we choose to model second-order beliefs as a **unimodal beta distribution**. The beta distribution is often encountered as the conjugate prior for a Bernoulli random variable. For example, when trying to estimate the bias of a coin with a completely uninformed prior, after observing *n* heads and *m* tails the posterior over the value for the bias of the coin is given by B(*n* + 1, *m* + 1). The mode of this distribution $\frac{n}{n+m}$ is one salient point estimate for the bias.

Conventionally, the values *n* + 1 and *m* + 1 are written as *α* and *β* respectively when they are used as parameters for the beta distribution. *α* and *β* need not be integers, but can be positive reals. We obtain a **unimodal** beta distribution when *α*, *β* > 1. In terms of these parameters the probability density function is given by:

$$\text{B}\left(\alpha ,\beta \right)≔x^{\alpha -1}{\left(1-x\right)}^{\beta -1}\cdot \frac{\Gamma \left(\alpha +\beta \right)}{\Gamma \left(\alpha \right)\Gamma \left(\beta \right)},$$

where Γ is the Gamma function. When convenient, we use an alternative parameterization of the beta distribution in terms of its *mode ω* and *concentration κ*:

$$\omega ≔\frac{\alpha -1}{\alpha +\beta -2},\;\kappa ≔\alpha +\beta .$$

**Figure 2.**
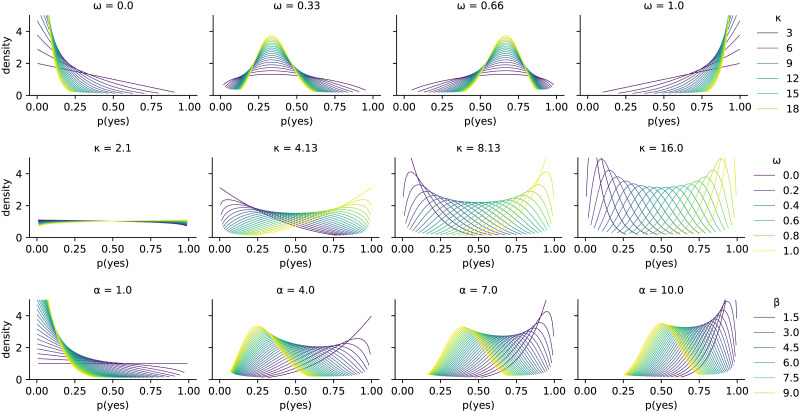
Illustration of beta distributions with each facet showing a fixed mode *ω* with varying concentrations *κ* (top row), or a fixed concentration with varying modes (middle row), or a fixed *α* with varying *β*.

This parameterization is often preferable because it more closely resembles the judgments that we measure experimentally (see Experiment 1: Individuals’ Beliefs Predict Relevance Judgments section). Intuitively, *ω* corresponds to the point probability that one might choose if forced to express their first-order beliefs, while *κ* is correlated to how peaked one’s second-order belief state is around that point estimate. For the sake of illustration, Figure 2 shows how these two parameters independently affect the shape of the beta distribution.

## CANDIDATE MEASURES OF EPISTEMIC RELEVANCE

**Figure 3.**
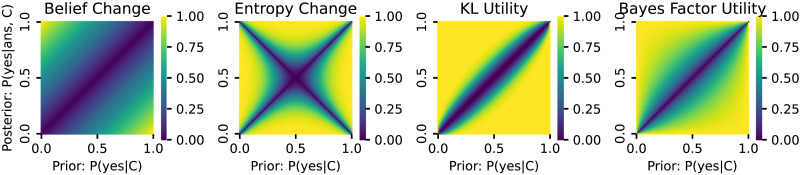
Visualization of the four first-order measures we compare. The *x*- and *y*-axes show the prior and posterior probabilities for the ‘yes’ answer, and shading shows the measure’s value.

**Figure 4.**
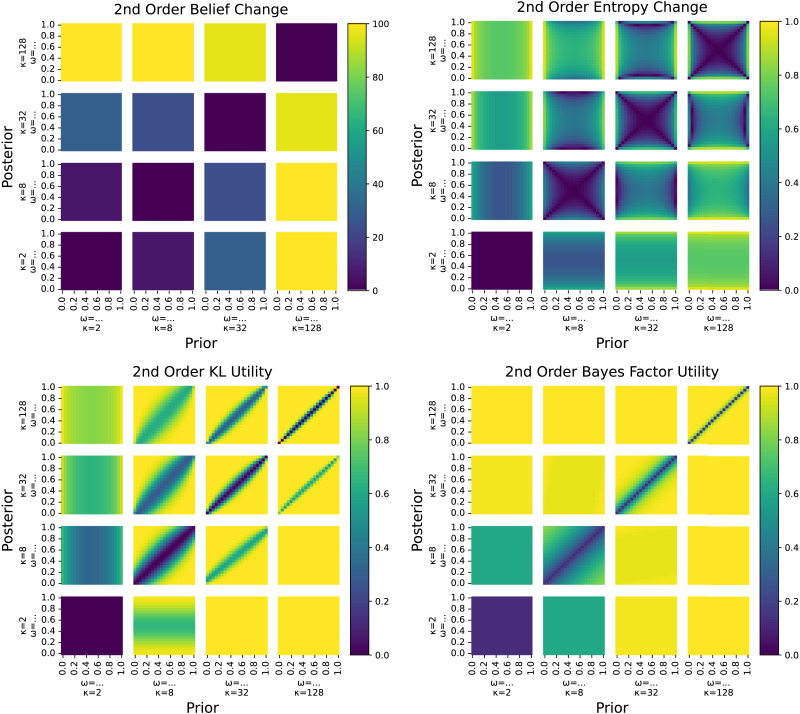
Visualization of the four second-order measures we compare. For each small heatmap, the *x*- and *y*-axes show different values of the prior and posterior beta distributions’ modes *ω* with fixed concentrations *κ*. Different heatmaps show different values of the prior and posterior’s concentrations. Shading shows the measure’s value.

The prior literature surveyed in Utility, Relevance, and Information section suggests several possible measures of epistemic relevance. Most salient for linguistic analysis are quantitative measures based on information-theoretic notions like entropy change or Kullback-Leibler divergence (KL divergence), and Bayesian measures derived from the likelihood ratio (Bayes factor). All of these notions essentially compare a prior belief (before hearing the answer) against a posterior belief (after processing the answer), but they have different conceptual motivation. Entropy change measures the change in uncertainty associated with the update. KL divergence measures the excess cost of using a code optimized for the prior rather than the posterior. Bayes Factor utility measures the strength of the evidence provided by the response in favor of a particular alternative. Following the arguments from Utility, Relevance, and Information section, we consider both first- and second-order beliefs. On top of theoretically motivated measures, we also consider *pure measures* computed directly from the data observations from our experiment. Table 1 below summarizes all measures introduced in the following, and Figures 3 and 4 visualize all first- and second-order measures, respectively, as a function of the prior and posterior. In the following, *p* and *q* are the prior and posterior probabilities of a ‘yes’ answer, **p** = 〈*p*, 1 − *p*〉 and **q** = 〈*q*, 1 − *q*〉 are corresponding first-order prior and posterior beliefs over both ‘yes’ and ‘no’ answers. We use *c* and *d* for the commitment levels for the first-order prior and posterior beliefs. For second-order beliefs, the pair *α*_0_ and *β*_0_ represents the parameters of the beta distribution for the prior beliefs, while *α*_1_ and *β*_1_ represent the corresponding posterior beliefs.

**Table 1.** Overview of candidate measures of relevance. Here, probabilities *p* and *q* are the first-order prior and posterior beliefs in a ‘yes’ answer, respectively, and Likert scale values *c* and *d* are commitment levels for the second-order prior and posterior beliefs. The vectors **p** and **q** are the first-order beliefs (assigning probability to both ‘yes’ answer and ‘no’ answer). The parameters for the beta distribution *α*_0_ and *β*_0_ describe the second-order prior beliefs, while the pair *α*_1_ and *β*_1_ describes the second-order posterior beliefs. *g_*r*_(*x*) = 1 − *r*^−*x*^. **a = −1 if *p* > *q* and *a* = 1 otherwise.

**Family**	**Measure Name**	**Order**	**Definition**
Direct	probability change	1st	|*p* − *q*|
	commitment change	2nd	|*c* − *d*|
	concentration change	2nd	*g*(|*α*_0_ + *β*_0_ − (*α*_1_ + *β*_1_)|)*
Entropy	entropy change	1st	*g*(|H(**q**) − H(**p**)|)
	second-order entropy change	2nd	*g*(|H(B(*α*_1_, *β*_1_)) − H(B(*α*_0_, *β*_0_))|)
KL divergence	KL utility	1st	*g*(KL(**q**‖**p**))
	second-order KL utility	2nd	*g*(KL(B(*α*_1_, *β*_1_)‖B(*α*_0_, *β*_0_)))
Bayes factor	Bayes factor utility	1st	1 − BF_*polar*_(*p*, *q*)^a^**
	second-order Bayes factor utility	2nd	1 − *g*(log(|*α*_1_ − *α*_0_| + |*β*_1_ − *β*_0_| + 1))

We add the restriction that all measures we consider are bounded, usually on the interval [0, 1] or [0, 1) While all underlying information-theoretic notions we consider are already bounded below by zero (inclusive), many are unbounded above. To these measures, we apply a transformation function *g*(*x*) = 1 − *r*^−*x*^, where *r* is a free parameter, which maps the interval [0, ∞) to [0, 1). This has two purposes: First, it ensures in subsequent analysis that any differences between metrics are not due to the existence of an upper bound. Second, the resulting measures more closely resemble the human relevance judgments we gather, which are on the interval [0, 1] (see Experimental Design section).

In Pure Measures–Bayes Factor sections, we discuss the justification of each measure from the perspectives of Information Theory and Bayesian reasoning. In Worked Examples section, we discuss concrete of examples and compare the predictions of each measure.

### Pure Measures

To quantify first- and second-order belief change as directly from behavioral data as possible, we simply operationalize belief change as the absolute value difference in the relevant judgment from the prior to posterior. probability change, defined as |*p* − *q*|, is the absolute change in probability judgments for the positive answer from the prior to posterior. commitment change, defined as |*c* − *d*|, is the absolute change in the participant’s level of commitment to their probability judgment from the prior to posterior.

An alternative pure measure of second-order belief change is concentration change, which quantifies the change in those factors of second-order belief that are largely independent of first-order factors:

$$g\left(\left|\alpha_{0}+\beta_{0}-\left(\alpha_{1}+\beta_{1}\right)\right|\right).$$

We use *α*_0_ and *α*_1_ to denote the value of the *α* parameter in the prior and posterior, respectively, and similarly for *β* and other symbols. The above quantity is the (transformed) absolute value difference of the concentration of the prior and posterior beta distribution. If we interpret *α* and *β* as the counts of the outcomes of Bernoulli trials, then the concentration is always two less than the total number of trials, as explained in Representing Second-Order Belief States section. Thus, the concentration corresponds to the quantity of evidence without providing any information about which hypothesis the evidence favors. Outside the context of repeated Bernoulli trials, the concentration can either increase or decrease from prior to posterior.

### Entropy Change

Intuitively, entropy change measures the difference in uncertainty between two distributions. When applied to question-response pairs, entropy change is the change in an agent’s uncertainty before and after updating their beliefs based upon the response. More concretely, the Shannon entropy (Shannon, 1948) measures the expected surprisal over the outcomes of a random variable, or in our case the expected surprisal of the answer to the QUD. Formally, the entropy H of the distribution described by probability vector **x** = 〈*x*_1_, …, *x*_*n*_〉 is:

$$\text{H}\left(x\right)=-\sum_{i}x_{i}\log x_{i}.$$

The first-order measure of entropy change is defined as the difference in this quantity in the prior and posterior, which we scale using the transformation *g*:

$$g\left(\left|\text{H}\left(p\right)-\text{H}\left(q\right)\right|\right).$$

When updating with a response, the agent’s posterior distribution might become more uncertain (closer to uniform) or less uncertain. In this work we use entropy *change*—the absolute value of the difference—rather than entropy *reduction*—the raw difference. The raw difference is positive in cases where the posterior gains certainty (relative to the prior), and it is negative when the posterior loses certainty. While it might be intuitive to view answers that increase certainty as preferable, it is not reasonable for responses that decrease certainty (possibly due to unjustified prior beliefs) to be considered less relevant than responses that do not change beliefs at all. By measuring the absolute difference, we treat responses that increase certainty and responses that decrease certainty to the same degree as equally relevant. However, both entropy change and entropy reduction make the unintuitive prediction that belief change that preserves entropy while changing individual alternatives’ probabilities have zero utility. For example, suppose **x** assigns 0.1 probability to event *a* and 0.9 to *b*, while **y** assigns 0.9 to *a* and 0.1 to *b*. The change in entropy is 0 because the degree of uncertainty is unchanged, although how that uncertainty is distributed over alternatives has changed.

We can also lift entropy change to second-order beliefs. The differential entropy of a Dirichlet distribution *P*, of which the beta distribution is a special case, with parameters *α*_1_, …, *α*_*k*_ is given by:

$$\text{H}\left(P\right)=\log\text{B}\left(\alpha^{k}\right)+\left(\alpha_{0}-k\right)\psi \left(\alpha_{0}\right)-\sum_{i=1}^{k}\left(\alpha_{i}-1\right)\psi \left(\alpha_{i}\right),$$

where *α*_0_ = $\sum_{k=1}^{K}$ *α*_*k*_, B(*α*^*k*^) = $\frac{\prod_{i=1}^{k}\Gamma \left(\alpha_{i}\right)}{\Gamma \left(\alpha_{0}\right)}$, and *ψ* is the digamma function. Accordingly, second-order utility from entropy changes, second order entropy change is defined analogously to the first-order variant as the absolute difference between entropy of prior and posterior second-order beliefs.

### Kullback-Leibler Divergence

The next pair of measures we consider compute utility using the KL divergence of the posterior and prior distributions over possible answers. KL divergence (Kullback & Leibler, 1951) can be interpreted as the expected excess surprise when using an incorrect distribution (in our case, the prior) as a model for a true or more informed distribution (the posterior). The distributions are here expressed as two probability vectors, **x** and **y**, over the same sample space.

KL divergence can be defined as the difference of the entropy of the informed distribution **y** and the cross-entropy of **x** and **y**:

$$\text{KL}\left(y\|x\right)=\text{H}\left(y,x\right)-\text{H}\left(y\right).$$

While entropy measures the average surprisal of the outcomes of a random variable *p*, using probabilities from *p* itself as weights, cross-entropy (also denoted as H) also computes a surprisal average, but instead uses probabilities from a different distribution as weights:

$$\text{H}\left(y,x\right)≔-\sum_{i}y_{i}\log x_{i}.$$

Thus, cross-entropy measures one’s expected surprisal when observing the outcome of **y** while under the false belief that the true distribution is **x**. By subtracting H(**y**) from the cross-entropy H(**y**, **x**), the KL divergence measures only the *excess* uncertainty due to holding false beliefs.

We define KL utility in the question-response setting as the KL divergence of the posterior **y** and the prior **x**, transformed by the function *g*:

$$g\left(\text{KL}\left(q\|p\right)\right).$$

Analogously to KL utility, we can compute the KL divergence of two beta distributions to quantify the expected excess surprisal from assuming the second-order prior when the posterior is correct. As before, we apply the transformation function *g* to obtain the measure second-order KL utility. The beta distribution is a special case of the Dirichlet distribution with two parameters. The KL divergence of two Dirichlet distributions *Y*, *X* with parameters *α*_1_, …, *α*_*k*_ and *β*_1_, …, *β*_*k*_, respectively, can be computed as follows:

$$\begin{array}{l} \text{KL}\left(X\|Y\right)=\log\Gamma \left(\alpha_{0}\right)-\sum_{k=1}^{K}\log\Gamma \left(\alpha_{k}\right)\\ \;-\log\Gamma \left(\beta_{0}\right)+\sum_{k=1}^{K}\log\Gamma \left(\beta_{k}\right)\\ \;+\sum_{k=1}^{K}\left(\alpha_{k}-\beta_{k}\right)\left(\psi \left(\alpha_{k}\right)-\psi \left(\alpha_{0}\right)\right), \end{array}$$

where *α*_0_ = $\sum_{k=1}^{K}$ *α*_*k*_, *β*_0_ = $\sum_{k=1}^{K}$ *β*_*k*_, and *ψ* is the digamma function (Lin, 2016).

### Bayes Factor

The Bayes factor is the likelihood ratio for two competing hypotheses, *H*_1_ and *H*_2_. Given data *D*, it is defined as:

$$\frac{P\left(D|H_{1}\right)}{P\left(D|H_{2}\right)}.$$

Applying Bayes’ Theorem, we have:

$$\frac{\frac{P\left(H_{1}|D\right)P\left(D\right)}{P\left(H_{1}\right)}}{\frac{P\left(H_{2}|D\right)P\left(D\right)}{P\left(H_{2}\right)}}=\frac{P\left(H_{1}|D\right)}{P\left(H_{2}|D\right)}\frac{P\left(H_{2}\right)}{P\left(H_{1}\right)}.$$

This reformulation shows that we can think of the likelihood ratio as the factor by which the odds in favor of one of the two hypotheses change going from prior to posterior by updating with the data *D*. In this way, the Bayes factor is a measure of the weight of observational evidence (Good, 1950; Jeffreys, 1961; Kass & Raftery, 1995). It is a measure that does not depend on the (subjective) prior probabilities assigned to hypotheses, although it can be computed using the prior and posterior when the likelihoods cannot be directly measured.

In our application, the data *D* is the response to the polar QUD, and the competing hypotheses are the ‘yes’ answer and ‘no’ answer. The polar Bayes factor is thus

$${\text{BF}}_{\text{polar}}\left(p,q\right)≔\frac{q}{1-q}\frac{1-p}{p}.$$

Put intuitively, the polar Bayes factor measures how much the answer *A* favors the ‘yes’ answer interpretation over the ‘no’ answer. If the ratio is greater than 1, then *A* gives evidence in favor of a ‘yes’. If the ratio is less than 1 (it is always non-negative), this is interpreted as *A* giving evidence against a ‘yes’ answer. It is typical to take the log of the Bayes factor to obtain a value in the interval (−∞, ∞), where a value of 0 indicates no change from prior to posterior. If we do not care which alternative is favored, we can compare the absolute values.

If we further desire a utility score between 0 and 1, we can apply the transformation function *g*. However, *g*(|log(BF_*polar*_)|), can be simplified further. If the free parameter of the transformation function *r* is chosen as the base of the logarithm, we obtain the final simplified form of Bayes factor utility:^4^

$$1-{\text{BF}}_{\text{polar}}{\left(p,q\right)}^{a},\;\text{where}\;a=-1\;\text{if}\;p>q,\;\text{and}\;a=1\;\text{otherwise}.$$

Based on its behavior in the limit, we define this measure to equal 1 if either *p* or *q* equals 0 or 1, i.e., if either QUD is fully resolved. Furthermore, it equals 0 if and only if there is no (first-order) belief change.

The Bayes factor does not have a direct counterpart in the realm of second-order beliefs (because it is not clear how to relate it to a dichotomic issue). Therefore, we formulate a measure for second-order Bayes factor utility which captures the intuition of Bayes factor utility, namely to quantify the degree to which new observational evidence favors one hypothesis over another:

$$\left|\alpha_{1}-\alpha_{0}\right|+\left|\beta_{1}-\beta_{0}\right|+1$$

If we interpret the beta distribution as the conjugate prior of a Bernoulli random variable, then the quantity |*α*_1_ − *α*_0_| (similarly |*β*_1_ − *β*_0_|) measures the difference in the number of observations (counts of heads and tails), which would produce the prior and the posterior when starting from an unbiased, flat beta distribution B(1, 1). We add 1 so that, like the Bayes factor, this value is 1 when there is no belief change. As with the Bayes factor utility, it is reasonable to take the log of this quantity and apply the scaling function *g* so that the values lie on the interval [0, 1].

### Worked Examples

Table 2 gives several representative examples illustrating some of the key properties of different measures of relevance.

**Table 2.**
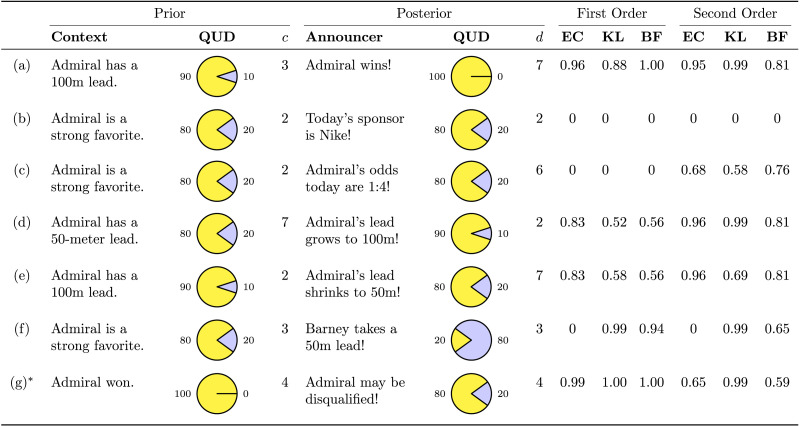
Hand-crafted examples of first- and second-order utilities in different scenarios with the QUD *Will Admiral win?*. Utilities shown are from the entropy change (EC), KL divergence (KL), and Bayes factor (BF) families. Pie charts show the probability that Admiral will win in yellow, and the probability that Admiral will not win in blue. *c* and *d* are prior and posterior commitment judgments on a 7-point Likert scale. Probabilities and commitment judgments are subjective judgments of the authors. Second-order beliefs are modeled as beta distributions with *ω* given by the probability that Admiral will win and *κ* given by applying the respective linking function in Table 11 to *c* and *d*. *The prior belief state in this example is not necessarily Bayesian. See discussion below.

#### Exhaustive Answer (A).

Example (a) is an exhaustive answer which fully resolves the QUD, i.e., all probability mass is on one alternative. Exhaustive answers, while considered maximally relevant under most logical approaches (Roberts, 2012), are only maximally relevant according to the Bayes factor utility. In fact, second-order Bayes factor utility also approaches its maximum as either *α* or *β* approaches infinity, i.e., when all probability mass is on either answer; however, we do not observe this as we place an upper bound on the concentration in practice (see Experimental Design section). For all other metrics, the degree of relevance depends on the degree of belief change.

#### Non-Answer (B).

Example (b) is a non-answer which does not alter the listener’s beliefs relative to the QUD, i.e., both first- and second-order distributions are identical in the prior and posterior. Prescriptively, an adequate measure of epistemic relevance should assign minimal relevance to such responses. All metrics have this property, assigning a value of 0.

#### Second-Order Belief Change Only (C).

Example (c) is response that alters second-order beliefs (in this case by increasing the concentration *κ*) but—under our subjective judgments—does not alter first-order beliefs or the mode *ω* of second-order beliefs. Such responses have zero relevance according to all first-order measures, and non-zero relevance for second-order measures.

#### Symmetry (d), (e).

Examples (d) and (e) have symmetrical prior and posterior, i.e., the prior of one is the posterior of the other. While entropy change and Bayes factor utility and their second-order counterparts are always symmetrical with respect to prior and posterior, KL utility and second-order KL utility are not. In particular, KL-based measures are higher when the prior is more extreme.

#### Swapped Probabilities (f).

Example (f) is a response that alters beliefs only by rearranging the alternatives. In first-order terms, this means the prior probability of the ‘yes’ answer is the posterior probability of the ‘no’ answer. In second order terms, it means that *α*_0_ = *β*_1_ and *α*_1_ = *β*_0_ (equivalently, *ω*_0_ = 1 − *ω*_1_ and *κ*_0_ = *κ*_1_). Prescriptively, the degree of belief change should determine the relevance. While this is true of most metrics, this example reveals a failure mode of entropy change and second-order entropy change: These metrics are 0 because the entropy remains constant. In general, when there are more than two alternatives, there can be infinitely many posteriors that differ from a given prior but have the same entropy.

#### Certain Prior (d), (g).

Examples (d) and (g) show different conditions under which some aspect of the prior but not the posterior is certain. Such a belief update is not necessarily rational: Under Bayesian reasoning, if the prior is certain, the posterior must be as well. These examples reveal a less intuitive property of KL-based measures: They are maximal only when the *prior* is certain, and not when the posterior is certain. In fact, they are technically undefined, but we can define them to have a value of 1 based on their limiting behavior. Bayes factor–based metrics are also maximal for the same reason as in (a), together with the fact that they symmetrical with respect to the prior and posterior.

In the case of (d), where only the second-order prior is certain (Note: this cannot be represented as a beta distribution) second-order KL utility and second-order Bayes factor utility are maximal, but KL utility and Bayes factor utility are not. As mentioned above, we have upper bounded the Likert scale for *c*, but these second-order measures approach 1 if this value is allowed to increase. There is a sense in which this second-order update is still Bayesian: the prior and posterior can represent one’s beliefs about their first-order belief states *at two different moments in time*. By contrast, in (g), only the first-order distribution is certain, thus only KL utility and Bayes factor utility are maximal. Second-order measures are not maximal because, although the mode *ω* is at 1, other probabilities have non-zero density. In all cases, entropy-based measures are not maximal.

## EXPERIMENT 1: INDIVIDUALS’ BELIEFS PREDICT RELEVANCE JUDGMENTS

Against this background, we assume that relevance is a gradient notion determined by some relationship between prior and posterior beliefs about a question under discussion. In our first and main experiment,^5^ we test the validity of this assumption and investigate further which notion or notions of utility guide pragmatic content choice and interpretation. Focusing on empirical data on intuitive relevance judgments for answers to explicit polar questions for a start, we refine this investigation by asking the following three questions:

**Research Question 1.1:** Is first-order belief change predictive of relevance?
**Research Question 1.2:** Is second-order belief change predictive of relevance?
**Research Question 1.3:** Which of the ways of measuring belief change are most predictive of relevance?

To address these issues, the experiment reported in this section is designed to collect data on prior and posterior beliefs—both first- and second-order—as well as intuitive judgments of relevance. Pilot Data section describes how data from a pilot study influenced our analyses and hypotheses. Experimental Design section describes the experiment. Fitting Free Parameters section explains how parameters for second-order beta distributions were obtained from participants’ choices. Results: Data-Quality Assessment section reports on rudimentary data analysis intended to test whether the data conforms to general expectations regarding the effect of the experiment’s main manipulations. The subsequent Quantitative Analyses section then addresses analyses that directly probe three research questions mentioned above. Exploratory Analyses section describes further exploratory analyses beyond the main hypothesis tests.

### Pilot Data

Data from a previous pilot study (*N* = 133) was available and guided the specification of exclusion criteria, statistical models, and the formulation of concrete research hypotheses. The data from the pilot study was not included in any of the final analyses. We used data from the pilot to optimize free parameters needed for computing some independent variables (e.g., parameters of estimated beta distributions, see Fitting Free Parameters section). The concrete formulation of the hypotheses is reproduced later, in Quantitative Analyses section, because it presupposes knowledge of the experiment.

### Experimental Design

#### Participants.

We recruited 279 participants through Prolific. Participants were paid $3.50 for completing the experiment. Participants could only take part once and had to satisfy the following criteria: English as first language, no language-related disorders, no participation in pilot experiment, previous approval rate: 100%, and participation in at least 100 previous studies on Prolific.

#### Materials.

Our stimuli consisted of 12 vignettes presenting brief dialogues consisting of a context, a question, and an answer (see Table 3). For each vignette, we constructed variants manipulating three factors. First, the factor ContextType has three levels (positive, neutral, or negative) and is intended to manipulate the prior beliefs about the probability of the ‘yes’ answer to the relevant polar question. Second, the factor AnswerCertainty has four levels (exhaustive, high-certainty, low-certainty, or non-answer) which reflect different degrees of evidence in the answer. Finally, the factor AnswerPolarity with two levels (positive or negative) distinguished positive and negative answers for all levels of AnswerCertainty except for non-answer.

**Table 3.** Examples of experimental stimuli. Every trial showed a context, which was intended to induce a certain prior bias (negative, neutral, positive) regarding the probability of a positive answer. After a question, there were different kinds of responses, designed to provide different degrees of evidence (exhaustive, high-certainty, low-certainty, non-answer) in favor of or against the ‘yes’ answer (positive, negative).

**Context**				
Negative bias		Neutral	Positive bias	
*You’re a burglar, trying to rob a house with your accomplice Chris. You’ve been staking out a good looking house for the last week, **and noticed that the family seems to have left for vacation.***		*You’re a burglar trying to rob a house with your accomplice Chris. You’ve been staking out a good looking house for the last week, **and noticed that the family often goes out to dinner around this time.***	*You’re a burglar trying to rob a house with your accomplice Chris. You’ve been staking out a good looking house for the last week, **and noticed that the family usually watches TV together around this time.***	
**Question**				
*Is there anyone inside the house?*				
**Response**				
	Exhaustive	High-Certainty	Low-Certainty	Non-answer
Positive	*The whole family is inside the house.*	*I heard voices coming from inside.*	*There’s a light on.*	*The house has two entrances.*
Negative	*The house is completely empty.*	*All the lights are off.*	*There are no cars in the driveway.*	

#### Procedure.

Each participant completed a total of 14 trials, of which 10 are *main trials*, 2 are *reasoning control trials* and 2 are *attention check trials*, which appeared in the same order for each participant (see Table 4).

**Table 4.** Sequence of experimental trials.

*Trial*	*Content*
1	main trial 1
2	reasoning control 1
3–4	main trials 2–3
5	attention check 1
6–7	main trials 4–5
8	reasoning control 2
9–12	main trials 6–9
13	attention check 2
14	main trial 10

The 10 main trials were sampled randomly without replacement from 12 vignettes. Each participant saw at least two exhaustive, two high-certainty, two low-certainty, and one non-answer trial. The remaining three answer conditions were sampled uniformly without replacement. For exhaustive, high-certainty, and low-certainty answers, at least one had a positive value and one had a negative value for AnswerPolarity. There was no variation in AnswerPolarity for non-answer trials. For the variable ContextType, every participant saw at least three instances of negative, neutral, and positive each. The level of ContextType for the last, tenth, trial was sampled uniformly at random. ContextType conditions were sampled independently from AnswerCertainty and AnswerPolarity conditions.

**Figure 5.**
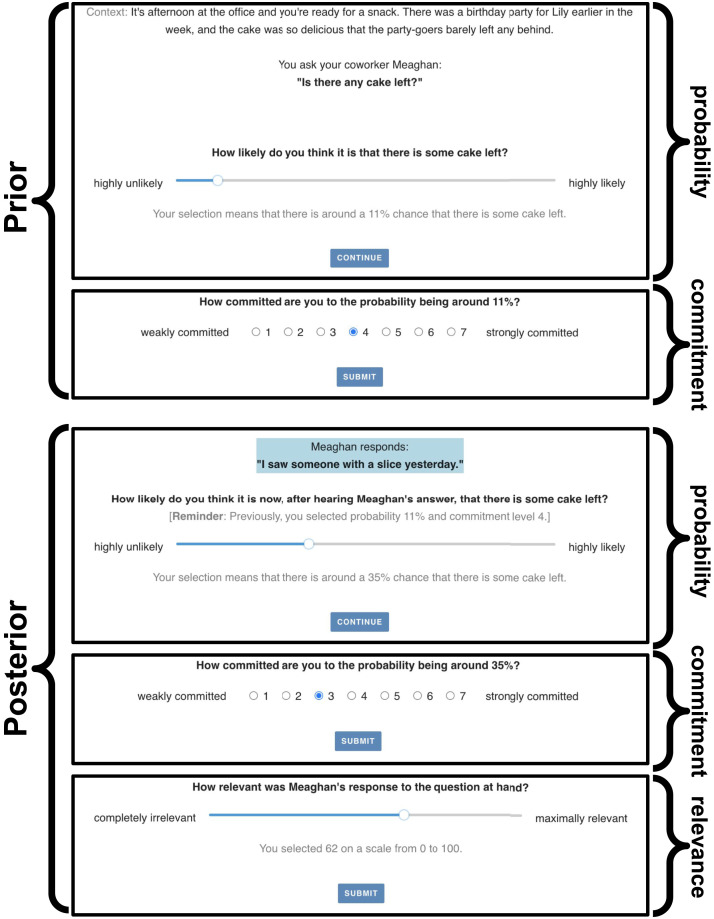
Screenshots from the five stages of a main trial from the experiment.

A single main trial was structured as follows (see also Figure 5). Participants first read the context description without the answer. They then indicated, using a slider, how likely they thought the positive answer was. Subsequently, they were asked to express their commitment to the indicated probability on a 7-point Likert scale. Next, they also read the question and were asked to, again, give a probability judgment with a corresponding commitment rating. Finally, participants were asked to judge the relevance of the answer, using a slider.

#### Notation.

Each trial then gives us a quintuple of data: 〈*p*, *c*, *q*, *d*, *r*〉, namely the prior probability judgment *p*, the prior commitment rating *c*, a posterior probability judgment *q* with its commitment rating *d*, and a relevance judgment *r*. The first-order prior distribution over positive and negative answers is identified directly from each participant’s posterior belief judgment, and denoted as **p** = 〈*p*, 1 − *p*〉. The same holds for the posterior distribution, which we denote as **q** = 〈*q*, 1 − *q*〉.

#### Attention and Reasoning Checks and Participant Exclusion.

Each participant also completed four filler trials which serve as quality checks. Participants were told at the beginning of the experiment that these checks will appear during the experiment. Two filler trials are attention checks, which instruct participants to ignore the vignette and select specific probability and commitment judgments. Each attention check trial provides two judgments 〈*p*, *c*〉. The remaining filler trials are reasoning checks, which assess participants’ first- and second-order probability judgments in simple unambiguous scenarios. Each reasoning check trial provides four judgments 〈*p*, *c*, *q*, *d*〉.

### Fitting Free Parameters

To compute measures of relevance, we must fit several free parameters. First, for each unbounded measure, we fit the parameter *r* for the scaling function *g*(*x*) = 1 − *r*^−*x*^. For each first-order measure, we optimize *r* to minimize the mean squared error of the measure and the relevance judgment over all the trials in the pilot study.

Second, for each second-order measure, we associate the participants’ prior and posterior belief states with a second-order distribution over probability values. This involves fitting additional parameters for each measure, which we do jointly with *r* using the same objective function as above. As discussed in Representing Second-Order Belief States section, we operationalize second-order belief states as unimodal beta distributions, parameterized by the mode *ω* and concentration *κ*. We derive these parameters from pairs of measurements of probability and commitment, respectively. For exposition, we use the information about the prior, *p* and *c*, here; the case for the posterior is analogous.

We equate the mode *ω* with the participant’s point probability judgment *p*. We based this decision on the assumption that, when forced to give a point judgment, the best guess is naturally represented by the value with the greatest probability density. We calculate the concentration as *κ* = *a* * *b*^*c*^, where *c* is the prior commitment rating and *a* and *b* are free parameters.^6^ Note that *a* and *b* are not the parameters of a beta distribution, which are instead notated as *α* and *β*.

The optimized parameters are given in Table 5. We optimize parameters individually for each measure in order to give every measure its greatest chance at explaining the data, whereas optimizing parameters jointly could unfairly advantage a subset of measures. The optimal values are usually fairly similar across measures, with a few exceptions: First, *r* is orders of magnitude larger for entropy change and KL utility than for other measures. This is not a concern because there is no reason to expect that the raw values of these metrics prior to scaling should have similar distributions. Second, the values of *a* and *b* are slightly larger for second-order Bayes factor utility than for other second-order measures. This may raise some concern as second-order belief states are not conceptually tied to any particular measure. However, we informally explore jointly optimizing parameters for all measures, and we find that doing so increases mean squared error by no more than 0.07 for any measure, suggesting that while the optimal parameter settings may differ between measures, different measures do not require substantially different second-order belief states to align with relevance judgments.

**Table 5.** Best-fit parameters for the linking function *κ* = *a* * *b*^*c*^ and the scaling function *g*(*x*) = 1 − *r*^−*x*^.

**Measure**	*a*	*b*	*r*
entropy change	–	–	1154.73
KL utility	–	–	885603.86
concentration change	1.49	1.52	1.19
second-order entropy change	2.00	2.01	2.18
second-order KL utility	2.09	1.89	2.73
second-order Bayes factor utility	3.20	5.19	1.14

### Results: Data-Quality Assessment

All data points were excluded from any participant who met any of the following criteria: (i) a less-than-perfect score for the attention checks (2 trials) × (probability, commitment); (ii) a score below 50% on reasoning checks (2 trials) × (prior, posterior) × (probability, commitment); (iii) a task sensitivity of 0.75 or lower, where task sensitivity is defined as the percentage of trials (excluding non-answer) where (a) prior-posterior change was 0.05 or less, or (b) there was non-zero change in commitment. From the 279 participants who took part in the experiment 59 were excluded by our exclusion criteria (12 due to failure of the attention check, 32 due to reasoning, and 15 due to insufficient task sensitivity (if applied in this order)). This resulted in data from 220 participants for analysis.

Our stimuli were constructed to vary prior probability, answer polarity, and answer strength by manipulating the experimental factors ContextType, AnswerPolarity, and AnswerCertainty. Before addressing specific research questions about measures of relevance, we start with a general quality assessment of the data by verifying that our main manipulations had the intended effects.

#### Effects of ContextType on Prior Ratings and Prior Commitment.

**Figure 6.**
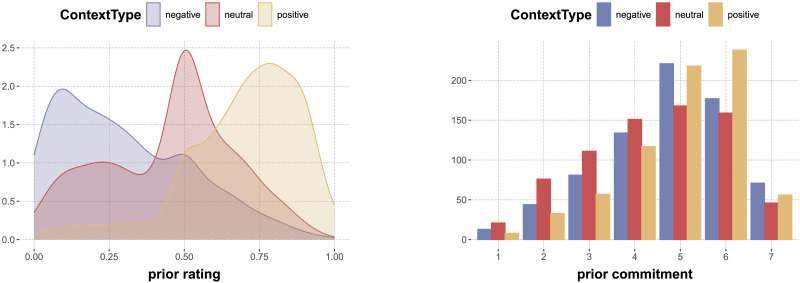
Effect of ContextType on prior probability judgments (left) and commitment judgments (right).

To check whether the ContextType manipulation worked, we compare participants’ judgments of the prior for each level of the ContextType variable. Visual inspection of the data (Figure 6, left) suggests that participants’ probability judgments for the ‘yes’ answer grouped by ContextType follow the expected order: negative < neutral < positive. Here and in the following, we analyze data from slider rating tasks with a Bayesian ordered beta regression model, using the R package ordbetareg (Kubinec, 2023). Hypotheses about ordinal effects of categorical factors are addressed by obtaining posterior samples under the default treatment coding of categorical variables, and then retrieving derived posterior samples for the differences between relevant combinations of factor levels with the help of the faintr package (Özenoglu et al., 2023).^7^ The first two lines of Table 6 show the results for the relevant ordinal comparison of prior ratings for different levels of ContextType. We find that the prior ratings in negative are credibly lower than in neutral, and credibly lower in neutral than in positive.^8^ So these results confirm that the ContextType manipulation worked as intended.

**Table 6.** Results from data-quality checks. For each of the contrasts in column “comparison” for the dependent variable in “measure”, the column “posterior” gives the estimated posterior probability that the relevant estimated means conform to the contrast in “comparison” in a Bayesian regression model predicting the “measures” (see main text for details on the models used). The last two columns give corresponding 95% credible intervals for each “comparison” (i.e., the interval for the difference between estimated means of the relevant design cells).

**Comparison**	**Measure**	**Posterior**	HDI-low	HDI-high
negative < neutral	prior	1	0.28	1.15
neutral < positive	prior	1	0.53	1.55
neutral < negative	prior-confidence	0.99	0.18	1.29
negative < positive	prior-confidence	0.8	−0.49	1.18
neutral < positive	prior-confidence	1	0.36	1.72

Figure 6 (right) also shows the distribution of (ordinal) commitment ratings for the prior task for different levels of ContextType. Visual inspection suggests that commitment in prior ratings may be higher in positive and negative than in neutral contexts. Here and in the following, we use Bayesian ordinal regression with a cumulative-logit link function to analyze data from the ordinal commitment rating tasks (Bürkner & Charpentier, 2020). Table 6 (last three lines) contains results of our exploratory analysis. As suggested by visual inspection, commitment ratings were credibly lower for neutral contexts than for either positive or negative contexts, but there is no indication for a difference between commitment ratings in positive and negative contexts. This finding is consistent with two interpretations: (a) probability and commitment do not vary independently in our stimuli, and (b) some participants misinterpret “commitment” to mean something like “level of commitment that the more probable answer is true”, as opposed to “level of commitment to the reported probability judgment”.

#### Effects of AnswerPolarity and AnswerCertainty on Changes in Beliefs.

To further check whether the experimental manipulations worked as intended, we define a simple intuitive notion of *directed belief change* and ask whether the manipulations of factors AnswerPolarity and AnswerCertainty had a reasonable effect on this measure. We define directed belief change as the difference between posterior and prior ratings in the direction expected from the answer’s polarity (i.e., belief change is positive if the rating for posterior beliefs in the ‘yes’ answer is higher than the prior rating, etc.). Concretely, if *p* is a prior rating and *q* the associated posterior rating, then directed belief change is defined as *a*(*q* − *p*), where *a* = 1 for positive answers and *a* = −1 for negative answers.^9^ For the current analyses we ignore non-answers. If our manipulations worked, we expect the following for both positive and negative cases of AnswerPolarity:

1. directed belief change is positive in each pair of levels from AnswerPolarity and AnswerCertainty; and
2. directed belief change increases from low-certainty to high-certainty, and again to exhaustive levels of factor AnswerCertainty.

**Figure 7.**
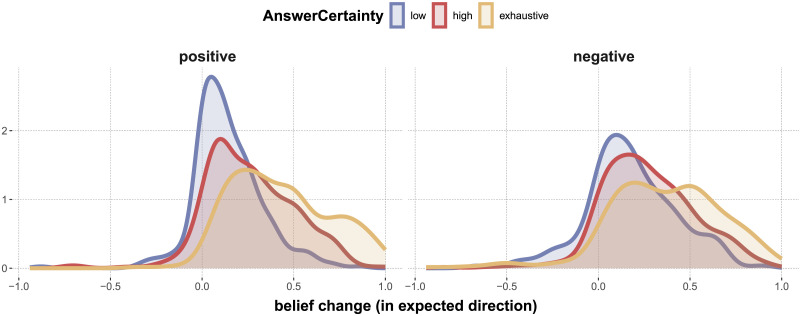
Distribution of directed belief change, defined as *a*(*q* − *p*), where *p* is the prior, *q* the associated posterior rating, and where *a* = 1 for positive answers and *a* = −1 for negative answers. Colors indicate different levels of factor AnswerCertainty, facets show different levels of AnswerPolarity. (Non-answers are excluded from the analysis for simplicity.)

Figure 7 shows density plots for our measure of directed belief change. Visually, the intuited properties seem to hold. We use a simple Bayesian regression model to address these exploratory hypotheses.^10^ We find that virtually all posterior estimates for all pairs of levels from AnswerPolarity and AnswerCertainty are positive, thereby supporting the first conjecture above. We also find support for the second conjecture in the results shown in the last two rows in Table 7. The first row in Table 7 also shows that there is no support for a difference in directed belief change when comparing just responses with positive and negative AnswerPolarity, which is reassuring as well.

**Table 7.** Results from comparing different factor levels with a simple Bayesian regression model predicting the measure *belief change*.

**Comparison**	**Measure**	**Posterior**	HDI (low)	HDI (high)
pos vs. neg polarity	belief change	0.46	−8.10 · 10^−2^	6.66 · 10^−2^
low vs. high certainty	belief change	1	6.59 · 10^−2^	0.17
high certain vs. exh	belief change	1	6.92 · 10^−2^	0.2

#### Effect of AnswerPolarity, AnswerCertainty, and ContextType on Relevance Ratings.

**Figure 8.**
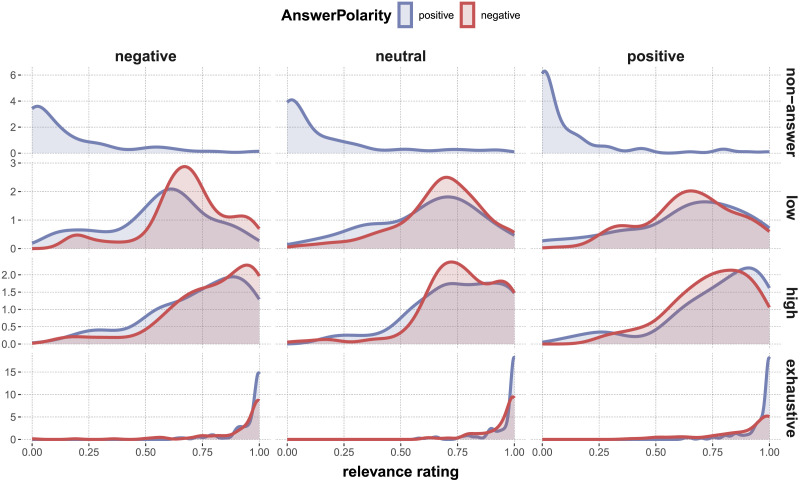
Estimated density of relevance ratings for different experimental conditions. The rows in the facet correspond to different levels of AnswerCertainty, the columns to ContextType. Colors distinguish AnswerPolarity (non-answers are treated as positive for convenience here).

Figure 8 shows the distribution of relevance judgments for different combinations of the relevant experimental factors. Results in Table 8 from an ordered beta regression model addressing a number of interesting comparisons (exploratorily) suggest that the more information an answer contained (non-answer < low-certainty < high-certainty < exhaustive), the more relevant the answer was rated, which is intuitive and suggestive that our materials and experimental manipulations had the intended effects.

**Table 8.** Results from exploratory testing the effect of experimental manipulations on relevance scores.

**Comparison**	**Measure**	**Posterior**	HDI (low)	HDI (high)
non-answer < low certainty	relevance	1	1.83	2.53
low certain < high certain	relevance	1	0.14	0.89
high certain < exhaustive	relevance	1	0.94	1.88
answer: pos < neg	relevance	0.43	−0.31	0.25
context: neutral > pos	relevance	0.99	1.82 · 10^−2^	0.32
context: neutral > neg	relevance	0.89	−5.36 · 10^−2^	0.22

### Quantitative Analyses

#### Research Question 1.1: First-Order Belief Change Explains Relevance Ratings.

A first important prediction about relevance tracking epistemic value is that higher belief changes (induced by the answer) lead to higher relevance ratings. Specifically, we test the hypothesis that the pure measure of probability change explains relevance ratings. We test this hypothesis by an ordinal beta regression model, using the R package ordbetareg (Kubinec, 2023), with maximal random effects that regresses relevance ratings against the absolute difference between prior and posterior ratings (probability change). As per a convention decided on prior to running our analysis, we judge there to be evidence in favor of this hypothesis if:

1. the relevant slope coefficient (for measure factor probability change) is estimated to be credibly bigger than zero (posterior probability > 0.944; an arbitrary value to indicate that there is nothing special about 0.95); and
2. a comparison with *leave-one-out cross validation* (Vehtari et al., 2017) of a model with an intercept only model substantially favors the model that includes the factor probability change.

The estimated posterior mean for the relevant slope coefficient is 2.948 (94.4% HDI: [2.261, 3.601]). The estimated posterior probability of the slope being positive is 1. The better model under leave-one-out (LOO) model comparison is the model *with* the factor probability change. The estimated log-probability difference for LOO model comparison is 348.89 (standard error of estimate: 23.872), which is significant under a simple *z*-score test, as suggested by Lambert (2018). From these results we conclude that the data provides evidence in favor of the hypothesis that relevance ratings are (partially) explained by first-order belief change.

#### Research Question 1.2: Second-Order Belief Change Additionally Contributes to Relevance Rating.

We hypothesize that change in commitment (factor commitment change) ratings additionally contributes to predicting relevance ratings. Concretely, we test this hypothesis with an ordinal beta regression model as described above, but also including the absolute difference in commitment ratings for before and after the answer (and the interaction term). We use the maximal random effects structure. We speak of evidence in favor of this hypothesis if the relevant posterior slope parameter is credibly bigger than zero and a LOO-based model comparison favors the more complex model. We speak of evidence against this hypothesis if the LOO-based model comparison favors the simpler model.

The estimated posterior mean for the relevant slope coefficient is 0.318 (94.4% HDI: [0.217, 0.417]). The estimated posterior probability of the slope being positive is 1. The better model under leave-one-out (LOO) model comparison is the model *with* the factor commitment change. The estimated log-probability difference for LOO model comparison is 53.071 (standard error of estimate: 11.137), which is a significant difference. From these results we conclude that the data provides evidence in favor of the hypothesis that commitment ratings contribute to predicting relevance scores additionally to the simple measure of probability change. In other words, higher-order uncertainty changes *do* seem to matter on top of first-order belief changes.

#### Research Question 1.3: Bayes factor utility Is the Best Predictor of Relevance Ratings.

**Figure 9.**
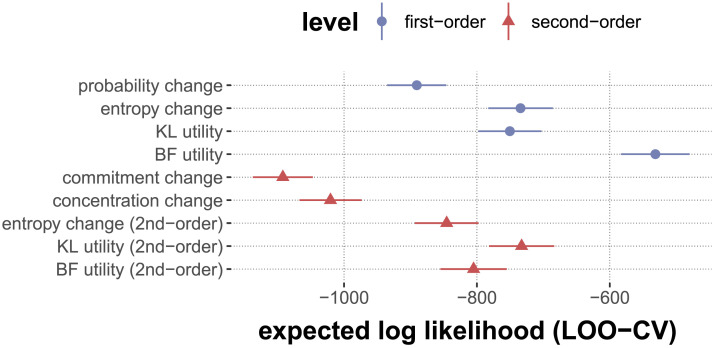
Results of comparing models with leave-one-out cross-validation. The models compared here are ordered beta regression models predicting relevance ratings with a single predictor (quantifying either first- or second-order belief change). Points and triangles show the estimated expected log likelihood for each held-out observation (under leave-one-out cross-validation). Larger values (further right) indicate a better fit. Line ranges indicate the standard errors for the estimates.

We also predict that our measure of utility of information derived from Bayes factors (factor Bayes factor utility) is a better (single-factor, linear) predictor of relevance judgments than the alternative measures. We test this prediction with LOO cross-validation. Figure 9 shows the estimated expected log-likelihoods for LOO-CV, visually comparing the performance of ordered beta regression models with single-factor predictor variables. The best model uses Bayes factor utility as the single predictor of relevance ratings.

When we directly compare Bayes factor utility to the second-best single factor predictor, second-order KL utility, the estimated log-probability difference for LOO model comparison is 201.404 (standard error: 20.814), which is a significant difference. This corroborates our hypothesis that, when using factors in isolation, the best predictor of relevance judgments is Bayes factor utility.

### Exploratory Analyses

#### Adding Second-Order Belief Change Improves Fit of All First-Order Models.

To complement research question 1.2, we also test whether adding another measure of higher-order uncertainty change adds predictive performance to each first-order measure of belief change. So here we compare, for each measure *X* (entropy change, KL utility, and Bayes factor utility) of first-order belief change, whether adding the factor concentration change increases the predictive performance. Concretely, we compare a model with a single factor *X* as a predictor to a model with the predictors *X*, concentration change, and their interaction. The latter model will be denoted *X* + SecondOrder for each *X*. For ease of fitting, no random effects are included.

In each case, the model with both first and second-order predictors performs better. First, we find that entropy change + SecondOrder beats entropy change, with an estimated log-probability difference of 38.241 (standard error: 9.062). Second, we find that KL utility + SecondOrder beats KL utility, with an estimated log-probability difference of 44.082 (standard error: 9.359). Third, we find that Bayes factor utility + SecondOrder beats Bayes factor utility, with an estimated log-probability difference of 23.155 (standard error: 6.43). In sum, it appears that for all three measures of first-order belief change, adding concentration change yields a substantially better model.

#### Among All Combinations of First- and Second-Order Measures, Combining Bayes Factor-Based Metrics Yields the Best Fit.

Finally, we compare ordered beta regression models with all combinations of first- and second-order measures. Questions of interest are:

1. Which arbitrary combination of first- and second-order measures is the best?
2. Does it matter to be consistent in the choice of first- and second-order measure. In other words, is the performance of “first-order X” always most boosted when we supply it with “second-order X” instead of some other “second-order Y”?

**Figure 10.**
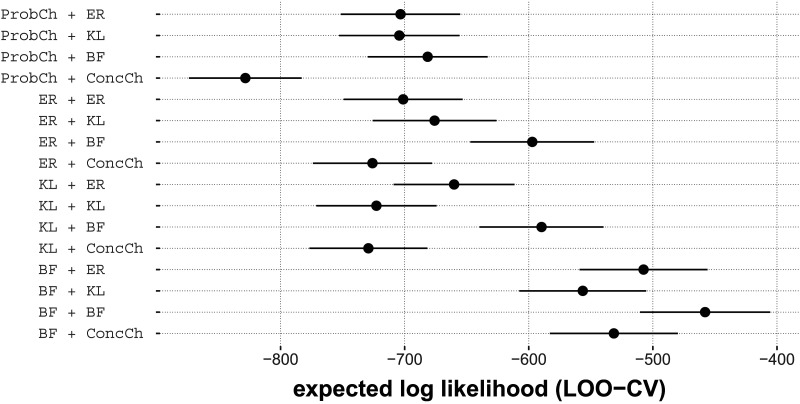
Results from LOO-CV model comparison of all ordered beta regression models freely combining first- and second-order predictors. Larger values (further right) indicate a better fit. The label *X* + *Y* refers to a model whose predictors are the first-order measure *X* and the second-order measure *Y*. The first-order and and second-order measures labeled pure are probability change and concentration change.

Figure 10 shows the results of LOO-based cross-validation for a plot comparing all possible combinations of first- and second-order predictors. By visual inspection, it seems that the overall best model in this comparison is the one that uses Bayes factor-based measures consistently. Indeed, the difference in expected log-likelihood between the best model (Bayes factor utility + second-order Bayes factor utility) and the second best model (Bayes factor utility + second-order entropy change) is 49.588 (standard error of estimate 12.965) and is significant by Lambert’s test. Moreover, Figure 10 shows that for each first-order measure *X*, the model ‘*X* + second-order Bayes factor utility’ is numerically better that any model ‘*X* + *Y*’ for any second-order measure *Y* other than second-order Bayes factor utility. To test whether this observation is substantial, we can compare the model ‘*X* + second-order Bayes factor utility’ to the second-best model ‘*X* + *Y*’ for *X*. We find that, for first-order measure probability change this difference is *not* significant (difference in expected log-likelihood 21.853, SE 23.014), *p* ≈ 0.877), but it is for all other first-order measures (entropy change: diff. 78.73, SE 17.684, *p* ≈ 0, KL utility: diff. 70.469, SE 18.01, *p* ≈ 0, Bayes factor utility: diff. 98.556, SE 13.777, *p* ≈ 0). Consequently, although the strong conclusion that second-order Bayes factor utility is the single *best* second-order measure to add is not supported (given the non-significant difference for probability change), we can conclude that it is not the case that “being consistent” (i.e., pairing each first-order measure with its corresponding second-order counterpart) is always best.

### Qualitative Analysis

Our experimental results quantitatively support our hypotheses that first- and second-order belief change both impact relevance, and that the Bayes factor utility is the best single statistic that predicts relevance from belief change. However, these results abstract away from particular examples which can provide insight into when relevance can be predicted on the basis of belief change, and when (and why) it cannot. For this reason, we conduct a qualitative analysis of the data. We begin by visually inspecting the predictions of each measure of relevance for every individual trial. This helps us extract case studies which are not easily explained by any measure of epistemic relevance. In General Discussion and Future Work section, we examine these case studies and use them to propose a typology of ways in which belief change and different interpretations of the notion of relevance diverge.

**Figure 11.**
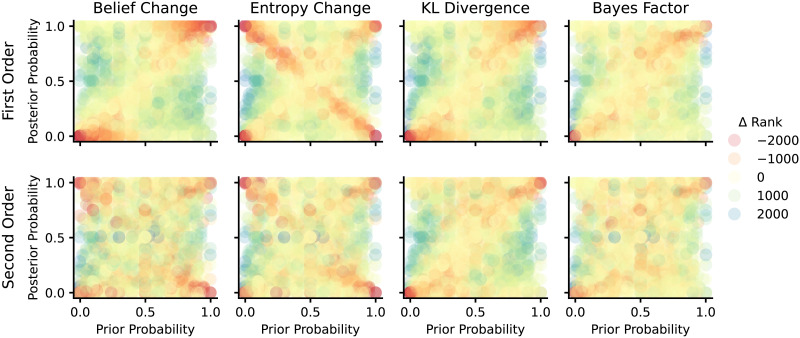
Each scatter plot shows for every trial how the predicted rank in terms of the given measure of relevance differs from its actual rank in terms of relevance judgment. Negative differences indicate that the measure under-predicts relevance. Within each scatter plot, points are arranged along the *x*- and *y*-axes by the participant’s prior and posterior judgments, respectively. Columns correspond to families of measures, while rows correspond to first-order and second-order counterparts of that family.

For each measure of relevance in our study, we examine the most and least extreme cases of divergence between the measure and the relevance slider ratings. Figure 11 considers only the *rank* of each data point (a given participant’s set of responses on a given trial) among all other data points in terms of the given measure, and plots how that rank differs from the point’s rank in terms of reported relevance. Better measures should have a rank difference near 0 for most data points, and points where a measure underpredicts relevance have a negative rank difference. Points are arranged on the scatterplot according to participants’ first-order prior and posterior probability judgments.

We observe that nearly all measures tend to underestimate relevance near the diagonal where prior probability equals posterior probability. This effect is strongest near the endpoints of the diagonal, where these values are close to 0 or 1. Agha and Warstadt (2020) made a similar observation regarding the first-order measures entropy change and KL utility, which they attribute to either confirmation bias or a failure to consider changes in second-order uncertainty. Indeed, Figure 11 shows that second-order measures tend to have less of a problem with underestimating relevance in these cases relative to their first-order counterparts.^11^

The Bayes factor-based measures (both first- and second-order versions) are least susceptible to underpredicting relevance along the endpoints of the diagonal. This is consistent with our finding that Bayes factor utility is the best predictor of relevance (Quantitative Analyses section). One intuitive explanation is that Bayes factor utility is the only measure that is maximal if either the prior or posterior is 0 or 1 (as long as the prior is not equal to the posterior). This is easily seen in Figure 3. This means that it correctly predicts maximal relevance for exhaustive answers even when the prior is highly biased. It also means that it can correctly predict high relevance in this region as long as the prior or posterior is near 0 or 1, even when probability change is small. (The reassuring phone call that you *did* win the Nobel prize is, presumably, highly relevant informational content, even though you were, modest as ever, highly certain that you would get it.)

Another general observation from Figure 11 is that measures tend to overestimate relevance when the prior is close to zero or one but the posterior is close to 0.5. This may suggest that participants reserve high judgments of relevance for exhaustive answers (i.e., when the posterior is 0 or 1), even if belief change is substantial. Once again, this failure mode is less extreme for Bayes factor utility when viewed in terms of the rank difference. This may come as a surprise, as Bayes factor utility actually always predicts maximal relevance if the prior is 0 or 1, while other measures do not (Figure 3). This counter-intuitive result is a consequence of focusing on the rank difference: Bayes factor utility also predicts maximal or near-maximal relevance in a larger region than other measures, so while the predicted relevance for a point in this region may be over-estimated, its rank may be lower than expected. By contrast, this is an especially strong failure mode for entropy- and KL divergence-based measures (both first- and second-order): e.g., entropy change is maximal in any case where one judgment (prior or posterior) is 0.5 and the other is 1 or 0, and KL utility is maximal when the prior is 0 or 1.

## EXPERIMENT 2: INTROSPECTION ON BELIEF STATES AFFECTS RELEVANCE JUDGMENTS

In each main trial of our experiment, every participant provides a prior probability judgment, a posterior judgment, and a relevance judgment in that order. Our expectation is that individual participants would vary in their use of the slider, in their prior estimation, and in their degree of trust in the information provided by the response. These forms of variation are expected on the hypothesis that an individual’s own relevance judgments can be predicted by the epistemic utility of the response relative to their own belief state. In order to control for this participant-level variation, we collect prior, posterior, and relevance judgments from each participant, enabling a within-subjects comparison. Having chosen a within-subjects design, we elicit relevance judgments after prior judgments because in the prior stage it is crucial that the participant does not yet have access to the response text.

While associated within-subject measurements provide a strong statistical signal, a potential downside of this approach is that each participant’s relevance judgment may have been influenced by the act of making previous prior and posterior judgments. To complete the picture, we would ideally want to compare relevance judgments from our main experiment to a “relevance-only” version of the experiment, where participants do *not* also supply prior and posterior ratings.^12^ This section reports on the results of such a “relevance-only” follow-up.

We aim to answer two questions:

**Research Question 2.1:** Does prompting individuals to introspect on their beliefs influence which measures of epistemic relevance best model relevance judgments?
**Research Question 2.2:** Does prompting individuals to introspect on their beliefs influence their relevance judgments?

What should we expect from such a comparison? Our view is that relevance is multifaceted, and in different contexts different notions of relevance may predominate. The aspect of relevance that we model here is what we call *epistemic utility*, and in Non-Epistemic Factors Influencing Relevance Judgments section we distinguish epistemic utility from other non-epistemic factors that may play a role in relevance judgments. On this view, it is expected that different experimental designs could cause participants to operationalize relevance in different ways. For example, in our experiment, participants are primed to weigh epistemic utility more highly. A different setting might cause participants to ignore epistemic factors, or cause them to be uncertain how to judge relevance. If these considerations are correct, then seeing little or no difference in relevance ratings between the previous main experiment and a “relevance-only” follow-up might imply that epistemic utility is a rather prominent aspect of intuitive relevance judgments. On the other hand, seeing pronounced differences might mean that, without nudges towards epistemic utilities, other aspects of intuitive relevance judgments are prominent, too.

### Participants, Materials, and Procedure

A total of 151 participants were recruited on Prolific, using the same recruitment criteria as for the main experiments, but excluding participants who had also participated in the previous main experiment. Participants were paid £1.50, resulting in an average hourly compensation of £11.84. The materials and procedure were exactly the same as before, except that participants did *not* see the prior/posterior elicitation trials, nor the prior/posterior confidence judgment trials.

### Results

#### Data Quality Assessment.

The data exclusion criteria for the previous experiment would have resulted in the rather severe exclusion of 95 participants who got one of the two reasoning trials wrong. We therefore decided to relax exclusion conditions for the “relevance-only” experiment so as to only exclude participants who got both reasoning trials wrong. This difference in reasoning trial performance is partly due to the fact that we now have fewer reasoning judgments to evaluate without the added prior and posterior judgments. We can also speculate that the shorter, more repetitive experiment may have invited a shallower engagement, or that the data quality has deteriorated in the participant pool over time.

#### Research Question 2.1: Introspection on Belief States Does Not Affect the Relative Fit of Measures of Relevance.

To compare the fit of measures of relevance to judgments from the “relevance-only” experiment, we perform an item-level analysis in which we compute the Pearson correlation between (a) the mean relevance judgment for the item from the “relevance-only” participant pool and (b) each measure of relevance computed from the mean prior and posterior judgments for the item from Experiment 1. Unlike in Experiment 1, we cannot perform a within-participant analysis as we do not have prior and posterior judgments for the “relevance-only” participants. Correlations from Experiment 2 data for each measure are provided in Table 9 (rightmost column). We again find that the Bayes factor utility is the best measure, although under this analysis, its fit is on par with entropy change. Naïve measures such as probability change and commitment change continue to show the worst fits.

**Table 9.** Fit of different measures across Experiments 1 and 2. ELL is the expected log-likelihood under leave-one-out cross-validation (larger values indicate better fit). Corr. is the Pearson correlation between relevance judgments and measures. The numerically best values for each column are bold-faced.

**Order**	**Measure**	**Exp. 1 ELL**	**Exp. 1 Corr**	**Exp. 2 Corr.**
1st	probability change	−890.513	0.610	0.560
	entropy change	−734.368	0.886	**0.770**
	KL utility	−750.405	0.824	0.706
	Bayes factor utility	**−531.330**	**0.891**	**0.770**
2nd	commitment change	−1092.160	0.594	0.567
	concentration change	−1020.461	0.712	0.654
	second-order entropy change	−845.519	0.758	0.693
	second-order KL utility	−732.733	0.889	0.732
	second-order Bayes factor utility	−805.006	0.875	0.702

To address the question of whether the best measures of relevance differ across experiments, we compare the rankings of each measure’s fit in the two experiments. Rankings are determined by estimated log-likelihood difference for Experiment 1 (Table 9, leftmost column) and by Pearson correlation for Experiment 2 (Table 9, rightmost column). The Spearman correlation between these ranked lists is 0.933, suggesting that the change in experimental protocol had a minimal effect on the relative fit of the candidate measures.

Given the difference in analysis method between Experiments 1 and 2, we also conduct a comparable item-level analysis using data from Experiment 1. We compute the Pearson correlation as above between the mean relevance judgments from Experiment 1 and the measures of relevance computed from Experiment 1 data. This gives us two ranked lists of measures according to Pearson correlation, for Experiment 1 and 2 relevance judgments. The fit of measures is consistently greater using relevance judgments from Experiment 1. Performing the same comparison of rankings as before, the Spearman correlation is 0.95, suggesting that neither the change in experimental protocol nor in analysis technique greatly influences our broader conclusions about the ranking of relevance measures according to their predictive potential.

#### Research Question 2.2: Introspection on Belief States Affects Relevance Judgments.

**Figure 12.**
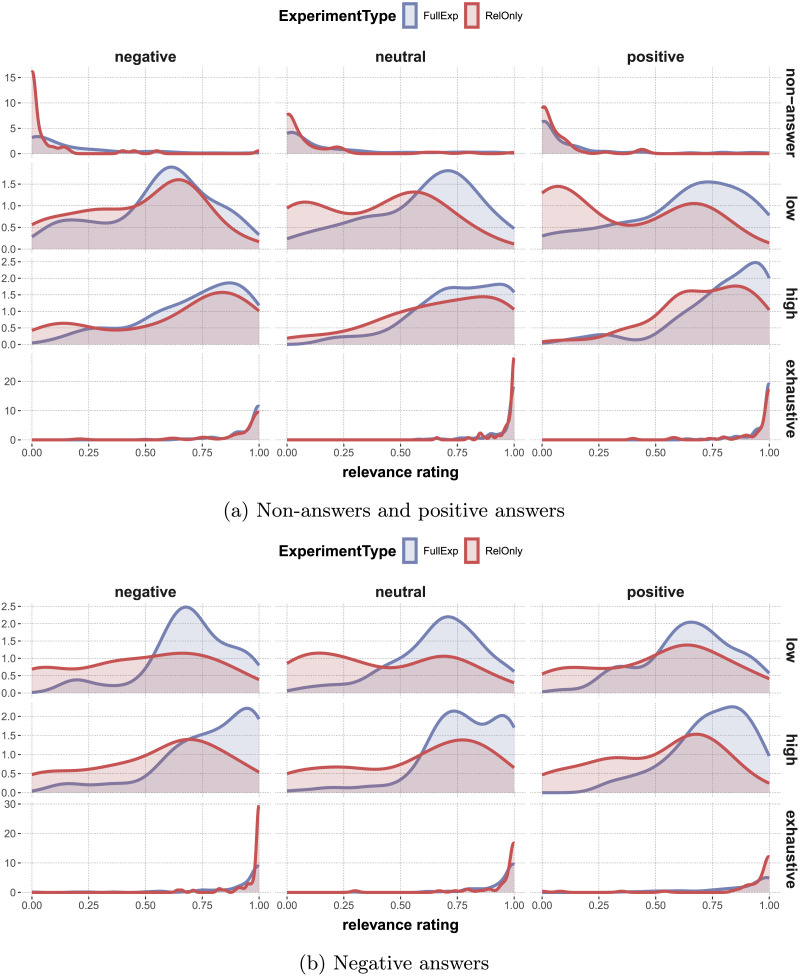
Distribution of relevance ratings in the main experiment (blue lines) and the “relevance-only” replication (red lines). Figure 12a shows the results from the non-answers and positive answers conditions, while Figure 12b shows the results from the negative answers. Columns show different levels of ContextType, and rows show different levels of AnswerCertainty.

Next, we compare results from Experiments 1 and 2 to test whether introspecting on belief states has *any* effect on relevance judgments (Research Question 2.2). Figure 12 shows the distribution of relevance ratings. Each facet shows the distribution of ratings in a different trial condition, while the red and blue density plots compare judgments from the main experiment and the “relevance-only” experiment. We expect to see certain patterns, such as low relevance ratings for low-certainty answers. Even by visual inspection, the distribution of relevance judgments for the “relevance-only” experiment seems to be more diffuse, often showing strong tendencies towards lower relevance judgments. Superficially, this supports the idea that participants may not have been as sensitive to the aspect of “epistemic utility” in their relevance judgments, possibly because they did not process the answers as deeply as when prompted explicitly about their beliefs and confidence.

To more precisely address the question of whether relevance judgments differ between experiments, we compare two Bayesian linear regression models. First, the baseline model regresses all relevance judgments from both experiments against all factors ContextType, AnswerPolarity, AnswerCertainty, their interaction and the maximal random-effects structure for items and participants. Second, the full model additionally included the factor ExperimentType and all main-factor interactions. This last factor indicates which experiment each data point was from. Comparing these two models with leave-one-out cross-validation, we find that the full model is significantly better than the baseline model (estimated log-probability difference 31.5 with estimated standard error of that estimate 12.2).

Results from model comparison suggest that the distribution of relevance judgments is different enough to require the inclusion of the variable ExperimentType for predictive accuracy. This corroborates the visual impression from above: Relevance judgments do seem to be distributed differently in the two experimental variants.

#### Discussion.

Results from Experiment 2 relativize, but do not undermine, our conclusions from the Experiment 1. All experiments and analyses indicated that the Bayes factor utility was the strongest measure of relevance that we tested. However, participants’ relevance judgments did differ between the two experiments, and in particular, for all epistemic measures of relevance, relevance judgments from Experiment 1 were better aligned. We cannot rule out that this effect is due to changes in the Prolific participant pool between the two experiments (which were run over 1 year apart), due to the shorter trials encouraging shallower engagement, or due to the fact that normal individual differences in relevance judgments and probability judgments are minimized only in Experiment 1 as a result of coming from the same participants. Still, this finding suggests that prompting participants to explicitly reason about their change in belief state likely did impact their judgments of relevance.

It is debatable whether guiding participants in this way is a confound or a desirable feature. While our goal was to compare which of several candidate measures might best explain a notion of relevance addressable as “epistemic utility,” the colloquial notion of relevance is multifaceted with several complementary interpretations. A disadvantage of drawing participants’ attention to belief states is that the resulting relevance judgments do not represent the full range of intuitions about relevance. On the other hand, an advantage is that the judgments are less likely to vary due to conflicting understandings of relevance from participants, and they are more likely to reflect the theoretically well-defined notion of epistemic utility. As far as making a methodological recommendation, we conclude that when designing experiments, investigators should consider whether or not they wish to conflate potentially conflicting associations with the term “relevant.”

## GENERAL DISCUSSION AND FUTURE WORK

Our findings suggest some clear conclusions about epistemic relevance: first- and second-order belief change do drive judgments of epistemic relevance, and the Bayes factor utility is the best measure for capturing these judgments. However, there is a more complicated picture of relevance that we wish to discuss. In our discussion, we enumerate factors other than the asker’s prior and posterior that could affect judgments of relevance (Non-Epistemic Factors Influencing Relevance Judgments section). We propose topics for future work, including studying relevance in non-polar questions and generalizing the Bayes factor utility to handle such cases (Generalizing Bayes Factor section) and distinguishing between relevance and utility from the asker’s perspective versus the answerer’s (Asker vs. Answerer Utility section). Finally, we sketch possible future experiments (Experimental Tests of Other Notions of Relevance section).

### Non-Epistemic Factors Influencing Relevance Judgments

In qualitatively analyzing our data, we observe numerous judgments of relevance which diverge from participants’ reported belief change, yet nonetheless seem consistent with some other reasonable interpretations of relevance on the naive participants’ part. Furthermore, the comparison between results in Experiments 1 and 2 (Table 9) suggests that prompting participants to explicitly reason about their belief states improves the fit of epistemic models of relevance to their introspective relevance judgments. While we do not know our participants’ justifications for their judgments, speculating about their reasoning has two benefits: First, it helps us identify possible sources of artifacts in our experimental data, where judgments systematically differ from our expectations. Second, it suggests other valid notions of relevance that require a different operationalization from our intended notion of epistemic relevance and which might be discouraged by prompting participants to reason epistemically. Through this qualitative analysis, we propose that different notions of relevance diverge in whether they consider the following variables: (a) the asker’s broader goals, (b) the asker’s prior beliefs, (c) the asker’s preferences, (d) the answerer’s credibility, (e) the answerer’s intent, and (f) the answerer’s completeness.

#### Asker’s Broader Goals.

In our operationalization of epistemic relevance, we have always equated the asker’s goals with the content of their question. The reality can differ from this idealization in two main ways: First, askers might have other epistemic goals, represented for instance as other questions under discussion lower on the QUD stack (Roberts, 2012). Second, askers might have non-epistemic goals. In either case, the existence of a broader goal might affect participants’ relevance judgments in our study. While we attempted to construct our stimuli to minimize obvious broader goals like this and instructed participants to judge how relevant the response was “to the question at hand”, we cannot totally prevent participants from inferring that there are broader goals.

The importance of broader goals to perceptions of relevance has long been known in pragmatics. For example, Clark (1979) showed experimentally that answerers change their responses to the same question (6) depending on the asker’s broader goal. Specifically, in response to (6-a), they found that liquor store employees were more likely to respond with the actual price, while in response to (6-b), they were more likely to respond ‘yes’ or ‘no’.

(6)  a. I want to buy some bourbon. Does a fifth of Jim Beam cost more than $5?
    b. I’ve got $5 to spend. Does a fifth of Jim Beam cost more than $5?

When it comes to the type of relevance judgments in our study, a broader goal might raise or lower the relevance of a response. The discourses in (7) provide examples where we have a literal QUD *Q* (*Does a fifth of Jim Beam cost more than $5?*) and a broader goal *G* (*To spend $5 at the liquor store*). In (7-a), the response has low relevance with respect to *Q*, but participants might judge its relevance to be high due to its practical relevance to *G*. In (7-b), the response’s low practical relevance to *G* might cause participants to assign it a low relevance score despite being exhaustive with respect to *Q*. By contrast, (7-c) leads to the same belief change with respect to *Q*, but provides additional information which increases the relevance to *G*.

(7)  [Context: I’ve got $5 to spend.]
    Does a fifth of Jim Beam cost more than $5?
    a. A fifth of Wild Turkey is $4.99.
    b. Yes.
    c. Yes, but a fifth of Wild Turkey is $4.99.

The full range of ways in which relevance is affected by the presence of broader goals is more complex than what this simple example illustrates. Furthermore, broader goals are often implicit and vague, so additional pragmatic inference is required to resolve them (Hawkins & Goodman, 2017; Stevens et al., 2016; Tsvilodub et al., 2023). Therefore, we leave further exploration of this rich topic to future work.

#### Asker’s Prior Beliefs.

**Figure 13.**
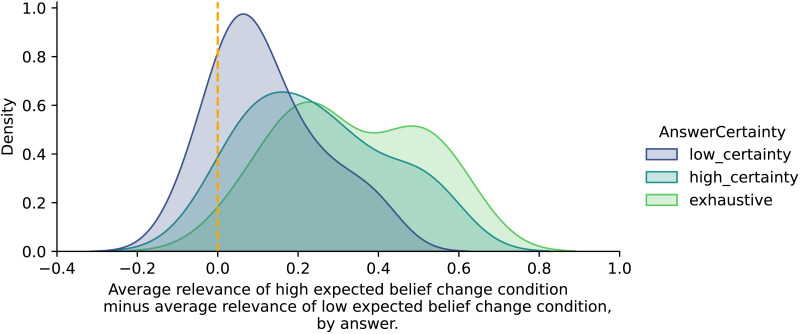
Impact of prior (ContextType) on an answer’s relevance. For each of the 72 answers in our stimuli excluding non-answers (3 AnswerCertainty levels × 3 AnswerPolarity levels × 12 vignettes), we compute the average relevance scores for that answer given contexts with positive and negative ContextType. If the answer has negative AnswerPolarity, we refer to the negative context as the lowExpectedBeliefChange condition, and the positive context as the highExpectedBeliefChange condition, and vice-versa for answers with positive AnswerPolarity. The plot shows the density of values for the difference highExpectedBeliefChange − lowExpectedBeliefChange across all 72 answers, separated by AnswerCertainty. Thus, if the prior context condition has the hypothesized effect on relevance judgments, we expect this difference to be positive.

Our measures of relevance all differ as a function of the asker’s prior beliefs. However, participants are often less sensitive to the prior than predicted: Figure 13 shows that a given answer is unlikely to be rated as more relevant when the context (and thus the prior) has the opposite AnswerPolarity of the answer. This is surprising because we do find higher probability change as an effect of context.

One explanation for this finding is that participants might be reasoning that the response *would be* highly relevant across a wide variety of contexts, even if it does not achieve high relevance in the particular context. In other words, participants might report the expected relevance score (e.g., Bayes factor utility) over all possible prior belief states.

Importantly, this explanation does not contradict our earlier conclusion that first-order belief change predicts relevance, though it does imply that belief change is not sufficient to explain relevance. Some answers may be associated with greater degrees of first-order belief change on average, meaning that across many different answers, the degree of belief change predicts relevance, even if it fails to do so for a given answer.

#### Asker’s Preferences.

In some cases, the asker may have an implicit preference for one QUD alternative over the other. In such cases, the asker might assign higher relevance to a response that favors the preferred alternative and assign lower relevance to a response favoring the dispreferred alternative, even if the latter has an equal or greater impact on the asker’s beliefs.^13^

One way for this effect to manifest would be that exhaustive answers could be assigned non-maximal relevance when they resolve the QUD to the dispreferred alternative. In our experiment, the asker’s preferred alternative would probably be determined by the vignette, so that different vignettes would lead participants to infer different goals and preferences. For example, in this vignette, the asker likely prefers a *no* answer.

(8)  Context: *Joe is the new guy at work, and he’s pretty cute. You’re thinking of asking him on a date, but if he’s married it would be pretty awkward, and you heard he has two children.*
    You ask your coworker Laura: *Is Joe married?*

While we did not find evidence that participants systematically report low relevance for exhaustive answers in certain vignettes, we cannot rule out that the asker’s preferences could, in principle, influence relevance judgments.

#### Answerer’s Credibility.

The same response may elicit different amounts of belief change depending on how credible the answerer is. Suppose a participant infers that the answerer is likely to be ignorant, mistaken, withholding of information, or deceptive. In that case, they should report less belief change than if the answerer is perfectly credible. However, when judging the relevance of the response, participants seem to differ in whether they consider this factor, or rather judge the response irrespective of whether it can be trusted. The latter interpretation can explain some cases in which participants attribute high relevance to a response, but report little belief change, as in (9).

(9)  Context: *It’s afternoon at the office and you’re ready for a snack. There was a birthday party for Lily earlier in the week, and the cake was delicious.*
    You ask your coworker Meaghan: *Is there any cake left?*
    Meaghan responds: *The cake box is still on the counter in the kitchen.*
    Prior: 0.63, Commitment: 3, Posterior: 0.76, Commitment: 4, Relevance: 0.84

The explanation is particularly compelling when answers are exhaustive, yet participants report less belief change than expected, as in example (10).

(10)  Context: *Joe is the new guy at work, and he’s pretty cute. You’re thinking of asking him on a date, but if he’s married it would be pretty awkward, and you heard he has two children.*
    You ask your coworker Laura: *Is Joe married?*
    Laura responds: *He’s single.*
    Prior: 0.74, Commitment: 4, Posterior: 0.25, Commitment: 6, Relevance: 0.99

As alluded to above, all of our operationalizations of relevance take the answerer’s credibility into account. This is because the asker’s posterior belief state depends on the answerer’s credibility. There are several ways to reformulate relevance that abstract away from answerer credibility. One option is to consider a maximally credible answerer, which appears to be what the participant in (10) has done. Another option is to take an expectation over a population of speakers with different levels of credibility. Either of these options grounds relevance in terms of belief change, except that belief change is computed in a counterfactual scenario with a different answerer or set of answerers.

#### Answerer’s Intent.

Another way in which belief change and judgments of relevance can diverge is when the asker infers that they do not fully grasp the answerer’s intended message. We most typically see this in the case of non-answers, where participants sometimes report little to no belief change, yet still judge the answer as relevant. The dialogue in (11) gives a concrete example.

(11)  Context: *It’s the day of Kim and Sam’s wedding. The wedding is at a beautiful beachside mansion. Last you heard, the plan was to have the ceremony on the beach, and the reception in the mansion ballroom.*
    You ask your friend Clara: *Will they have the ceremony outdoors?*
    Clara responds: *There are supposed to be 100 guests.*
    Prior: 0.81, Commitment: 6, Posterior: 0.80, Commitment: 6, Relevance: 0.45

One explanation for the participant’s judgments is as follows: The participant reasons that Clara intended for the response to trigger a **relevance implicature** (Grice, 1975) leading to a particular kind of belief change, but the participant lacks the necessary prior belief to make a belief update. For instance, if the participant had prior knowledge that the beach could only accommodate 50 guests, then their posterior probability judgment should be lower than their prior. But the update could easily go the other way if, for instance, they had prior knowledge that the indoor chapel was too small. Given this ambivalence, the participant is rational to stick to their priors.^14^ But crucially, their relevance judgment may reflect an inference that Clara must have been presupposing some more informed prior, and therefore she *intended* her response to be relevant.

Whether or not the answerer’s intent factors into the notion of “relevance“in this way, there may be individual variation across participants, leading to a possible confound in our results.

#### Answerer’s Completeness.

The asker might weigh the information provided by the answerer’s response against other possible information the answerer would know. This predicts that the same response would be judged highly relevant in a context where the asker thinks the answerer is telling everything they know, but less relevant in a context where the asker thinks the answerer is holding back.^15^

Some of our vignettes, such as the one below, are such that the answerer could plausibly have some motive to withhold information.

(12)  Context: *You and your colleague Lauren are CIA analysts. Lauren is a star employee with good credentials and a brilliant knack for puzzle solving. She seems completely loyal to the agency. On Monday your boss told you confidentially that your department is under investigation because the director suspects that a mole is leaking information to a foreign power.*
    You ask your colleague Alan privately: *Is Lauren the mole?*
    Alan responds: *Lauren is innocent, and she has a solid alibi.*

One potential analysis as follows: In addition to the asker’s probability distribution over QUD alternatives, they also have an expectation of informativity that varies according to the context, including what the asker thinks their interlocutor might know. If the asker’s expected informativity aligns with the informativity of the answerer’s response, then their judgment of relevance would likely behave according to our model predictions. But if the asker expects high informativity (perhaps suspecting a highly informed answerer), then relevance ratings might be deflated relative to the same answer in a context where they expect low informativity. In principle, quantitative models of relevance could be extended to take into account agents’ expectations about the informativity of future responses.

#### The Distinguished Role of Epistemic Utility.

In light of the existence of several valid but mutually exclusive notions of epistemic relevance, it may be better to refer to the particular notion we focus on in this work as **epistemic utility**. Clearly, a response’s actual utility for resolving a particular question in context *does* depend on the answerer’s credibility and the listener’s prior beliefs, and does not depend on other goals or on the speaker’s intent if that intent is not inferrable. In other words, a response’s epistemic utility depends only on the listener’s prior and posterior belief states.

While these alternate interpretations of relevance in some sense challenge our main conclusions from Quantitative Analyses section that belief change predicts relevance, they also serve to highlight the centrality of epistemic utility to the notion of relevance. With the notable exception of practical relevance, each alternative interpretation discussed above can be understood as adding something to our basic version of epistemic relevance as captured by a measure such as probability change or Bayes factor utility. These “additions“ range from computing the measure with respect to a broader QUD, or taking an expectation or maximum of the measure over a set of speakers or contexts. Thus, an adequate theory of information relevance as we have defined it is still necessary to give a theory of relevance more broadly.

### Generalizing Bayes Factor

A main limitation of Bayes factor utility is that it is only defined for QUDs with two alternatives. This limitation is not shared by other metrics, which are defined for QUDs with any finite number of alternatives. To address this, we suggest a simple extension to Bayes factor utility to QUDs with more than two alternatives (e.g., many *wh*-questions), which preserves nearly all of the desirable properties of the polar version. Specifically, we propose to take a weighted average of Bayes factor utility for each alternative in the QUD, weighted by the posterior probability. Given a question with *n* alternatives and **p** and **q** as prior and posterior probability vectors of length *n*, the generalized Bayes factor utility is

$$\sum_{i=1}^{n}q_{i}\cdot {\text{BF}}_{\text{polar}}\left(p_{i},q_{i}\right)=\sum_{i=1}^{n}q_{i}\cdot \left(1-{\left(\frac{q_{i}}{1-q_{i}}\frac{1-p_{i}}{p_{i}}\right)}^{a_{i}}\right),$$

where *a*_*i*_ = 1 if **p**_*i*_ > **q**_*i*_ and *a*_*i*_ = −1 otherwise.

This definition is a generalization of the one proposed in Bayes Factor section, i.e., it is equivalent for QUDs with two alternatives.^16^ Furthermore, this generalized measure maintains the property that it is equal to 1 if either the prior or posterior QUD is fully resolved (i.e., one alternative has probability 1).^17^ We leave empirical tests of this and other measures for QUDs with multiple alternatives to future work.

### Asker vs. Answerer Utility

Participants in our experiments inhabit the role of the asker (or listener). Future work might investigate whether answerers (or speakers) have a different way of computing relevance or utility.

This is important, for instance, in Rational Speech Act (RSA) models, where the speaker has a utility function that determines their chosen utterance. The speaker has a true answer in mind, and the utterance with the highest speaker utility is the one that minimizes the listener’s surprisal upon learning the true answer given the utterance. This notion of speaker utility is different from our notion of epistemic utility, which is only sensitive to the change in the listener’s subjective beliefs. Factors external to the listener, such as the speaker’s actual belief state or objective truth do not directly influence listener relevance.

Our epistemic utility also has no straightforward counterpart in RSA’s pragmatic listener. In standard RSA, the notion of relevance plays no role for the listener, who assigns credence to an alternative in proportion to the speaker’s utility of the utterance given that alternative. However, in some generative RSA models which output the pragmatic speaker’s distribution over utterances, the speaker chooses an utterance based in part on the epistemic utility under a listener (Merrill et al., 2022). Our work helps to refine what form this epistemic utility function should take. Furthermore, while prior work on probabilistic models of utterance choices in the RSA tradition has used mixture utility functions, combining informativity with other aspects of relevant language use, such as politeness (e.g., Yoon et al., 2020), practical relevance (e.g., Hawkins et al., 2025; Sumers et al., 2024), or closeness to the truth (e.g., Carcassi & Franke, 2023; Franke, 2014), one possibility which has not been explored is that speakers consider *both* speaker utility (in the RSA sense) *and* epistemic utility (in our sense). A mixed model like this would make quantitatively different predictions from the standard model. For instance, in the case of a highly confident listener, an exhaustive response will be low-utility under the standard model (as the listener already has low surprisal for the true answer), but high-utility under a model that incorporates Bayes factor utility. An important consideration is whether such models include a truthfulness component, as epistemic utility is not measured with respect to a single true answer.

### Experimental Tests of Other Notions of Relevance

In Non-Epistemic Factors Influencing Relevance Judgments section we described some intuitive relevance judgments that are not reducible to epistemic utility, and sketched a typology of different kinds of relevance. Future work could build on this typology by designing experiments to distinguish between epistemic utility and other notions of relevance. For example, epistemic utility could partially depend on the credibility of the speaker. It is also possible that utterances by highly credible speakers could be assigned high relevance even if those utterances do not have high epistemic utility relative to the listener’s prior, or that highly informative utterances by non-credible speakers could be assigned low relevance. To test this, information about the answerer’s credibility could be explicitly presented and manipulated, or the linguistic material in the answers could be systematically manipulated to study the effects of material that might signal the answerer’s credibility, e.g., through modal adverbs, evidential markers or miratives.

It may also be possible to find another dependent variable (besides relevance) that provides direct information about epistemic utility without requiring participants to provide explicit introspective judgments. For example, Herbstritt and Franke (2019) introduce an experimental paradigm that allows the manipulation of higher-order uncertainty without directly eliciting probability judgments. Participants imagine drawing colored balls from an urn and must guess the contents of the urn based on their sample. A similar task could be used to estimate the epistemic utility of answers, using their effect on participants’ guesses about concrete facts.

## CONCLUSION

The notion of epistemic relevance, formulated in terms of Questions Under Discussion, suggests several implementations in terms of Bayesian decision theory and information theory. While some of these implementations have been adopted in prior work, they have not been systematically evaluated against human data. Our work addresses this gap, experimentally evaluating four candidate models of epistemic relevance for both first-order and second-order beliefs by testing their ability to predict participants’ intuitions about the relevance of responses to polar questions. We found that the best predictor of these relevance judgments is based on the Bayes Factor—the ratio between the likelihood of the response given the ‘yes’ answer and the likelihood of the response given the ‘no’ answer. Intuitively, the Bayes factor measures how much the response favors the positive answer to the QUD over the negative answer.

The Bayes Factor, as the basis for a utility function, turns out to significantly outperform other *a priori* reasonable candidates, namely belief change (the absolute difference between the prior and posterior probability of *yes*), entropy change (the absolute difference between the entropies of the prior and posterior distributions), and KL divergence (specifically, the KL divergence between the posterior and prior distributions).

Moreover, we find that models are improved by taking into account agents’ commitment to their subjective probabilities. We treat an agent’s first-order beliefs as a categorical probability distribution over QUD alternatives. Second-order beliefs are therefore modeled as a distribution over these probabilities. Each measure above—Bayes factor, KL divergence, entropy change, and belief change—can be generalized to represent changes in second-order beliefs.

Among second-order predictors, the Bayes factor again has the best fit to human data, though the first-order Bayes factor remains the single strongest predictor. However, when second-order predictors are combined with first-order ones, the two Bayes factors together show the strongest ability to predict relevance judgments. Thus, no one measure on its own fully predicts relevance judgments, but human judgments consistently reflect Bayesian epistemic measures over common information-theoretic ones.

Finally, we suggest that measures like the Bayes factor model only a specific type of listener-oriented relevance, which we call epistemic utility. Other notions of relevance may be more appropriate for explaining some phenomena and may influence participants’ introspective judgments in experimental settings. We provide a framework to categorize other notions of relevance, and argue that many of these alternate notions can be defined in terms of epistemic utility.

Our work provides empirically supported recommendations to researchers in need of a measure of epistemic relevance in the service of some larger theory. It also sets the groundwork for a new research direction in cognitive science and pragmatics using empirical measurements of human relevance judgments to explain how humans select and evaluate utterances in discourse.

## ACKNOWLEDGMENTS

We thank Robert Hawkins and two anonymous reviewers for invaluable feedback on our work.

## FUNDING INFORMATION

The work of MF has benefitted from the project LMBayes (grant: Leibniz Collaborative Excellence 22-20, PI: Anton Benz).

## AUTHOR CONTRIBUTIONS

A.W.: Writing, experimental materials, running participants, data analysis. O.A.: Writing, experimental materials, data analysis. M.F.: Writing, running participants, data analysis.

## DATA AVAILABILITY STATEMENT

All experimental materials and anonymized participant response data are available at the following link: https://osf.io/7hyu6/overview?view_only=5d4f8d596d544b96a1f8e190cf04df92. Analysis code is available at the following link: https://github.com/magpie-ea/relevance-of-answers.

## Notes

^1^ Such answers may also be considered epistemically relevant with respect to a different question under discussion other than the current question.^2^ The criticism that relevance theory provides no quantitative measure of relevance had already been leveraged against the very first formulations of the research program (Gazdar & Good, 1982), and repeated again after subsequent key publications, for example by Levinson (1989) and multiple commentators of the position paper by Sperber and Wilson (1987) which accompanied the publication of the first edition of their book. The response by Sperber and Wilson (1995) is that relevance ought not be measurable, and that we should think of the definition in the quote above as an abstract characterization of the *kind of* mental calculation that the brain performs, not the actual calculation. Whatever the more general conclusions about the falsifiability of relevance theory as such should be, for our current purposes, this means that we cannot and probably should not use ideas from relevance theory, despite its name and centrality, to derive numerical measures of relevance for testing against our human data.^3^ If Jones is a Bayesian and updates a flat initial belief with the observed data, their best point-estimate will be *p* = 0.6, the mean of the posterior beta distribution with parameters 3 and 2. If Jones is frequentist, their best guess is a maximum likelihood estimate *p* = ⅔. If Jones adheres to any other (even more obscure) school of statistical reasoning, their estimate might be different again. For the example to serve as the intuitive argument, all that is required is that Jones is not absolutely sure about the value of *p*.^4^ If the base of the logarithm is *b*, we get 1 − BF_*polar*_(*p*, *q*)^*a* log_*b*_ *r*^, where *a* = −1 if *p* > *q*, and *a* = 1 otherwise. For simplicity, we let *r* = *b* in the remainder of the paper.^5^ We preregistered a variant of this experiment. Ultimately, our reported experiment deviates from the preregistered protocol due to methodological issues in the preregistered study which we later deemed to be minor but still worth addressing. Our study’s preregistration protocol is available (https://osf.io/7hyu6/overview?view_only=5d4f8d596d544b96a1f8e190cf04df92) along with stimuli, pilot data, and analysis scripts. A repository containing all final experimental data and analysis code is available (https://github.com/magpie-ea/relevance-of-answers). A description of the preregistered protocol and results from that analysis are available in Appendix A.^6^ We chose to link the commitment judgment and concentration by an exponential function because it generally gave better correlations with relevance judgments in the pilot data based on an informal comparison to other 2-parameter linking functions including linear and quadratic functions.^7^ All models are fitted with the maximum plausible random-effects structure licensed by the design, unless indicated otherwise, and all models used uninformative priors.^8^ For the current modest purposes, we speak of a credible difference if the posterior probability for the relevant difference measure is bigger than 0.944 (an arbitrary threshold, as a reminder that 0.95 is just as arbitrary), and the 95% credible interval for that parameter does not include zero.^9^ To avoid confusion, note that this is different from the previously discussion notion of probability change, which is defined as |*p* − *q*|.^10^ Although the dependent measure of directed belief change is bounded, we use a simple regression model here. While strictly speaking a wrong model, this is a mere sanity check, and we do not draw strong theoretically relevant conclusions from these analyses.^11^ We exclude commitment change from this analysis as the measure gives a small number of discrete integer values, so ranking trials is not particularly informative.^12^ We thank an anonymous reviewer for suggesting this experimental design.^13^ We thank an anonymous reviewer for suggesting this possibility.^14^ However, on this story, the participant perhaps should have decreased their commitment to the prior probability judgment. In general, though, this need not be the case.^15^ We thank an anonymous reviewer for this suggestion.^16^ An informal proof is as follows: When **p** and **q** have only two alternatives, BF_*polar*_(**p**_1_, **q**_1_) = BF_*polar*_(**p**_0_, **q**_0_). This follows because **p**_1_ = 1 − **p**_0_, **q**_1_ = 1 − **q**_0_, and *a*_1_ = −*a*_0_. Thus, both terms in the weighted sum are identical to the original Bayes factor utility, and their weights add to 1.^17^ An informal proof is as follows: As noted in Bayes Factor section, we define the Bayes factor utility to equal 1 when either *p* or *q* equals 0 or 1, and both are not equal. Thus, if one alternative in **p** or **q** is 1, all terms in the weighted sum equal 1, so the sum equals 1. The converse does not hold in general, but only fails pathological cases where alternatives that have zero probability in the prior have nonzero probability in the posterior, e.g., **p** = 〈0, 0, 0.5, 0.5〉 and **q** = 〈0.5, 0.5, 0, 0〉.^18^ https://osf.io/7hyu6/overview?view_only=5d4f8d596d544b96a1f8e190cf04df92.

## Figures and Tables

**Figure 14.**
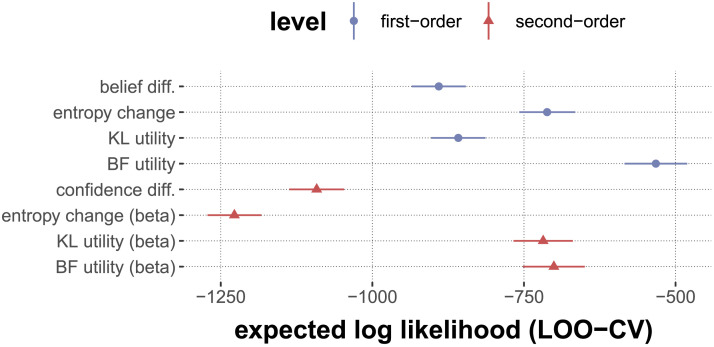
Results from the preregistered study of comparing single-factor models with leave-one-out cross-validation, analogous to the non-preregistered results in Figure 9.

## APPENDIX A: REREGISTERED PROTOCOL AND RESULTS

The methods and results presented in the main body of the paper differ in several ways from our preregistered protocol.^18^ This is due to several methodological choices in the computation of measures which, while not seriously problematic, we later deemed to be suboptimal, as the resulting measures were not all bounded. These changes do not affect the data collection procedure or statistical analysis. In keeping with best practices in open science, we also ran a version of the study exactly as preregistered, which we present below. The conclusions are not substantively different.

The preregistered measures are presented in Table 10. The differences are as follows:

1. The transformation function *g* is fixed to *g*_*e*_ (i.e., the parameter *r* is set to Euler’s constant *e*), rather than optimizing *r* for each measure.
2. The transformation function *g* is applied only to KL-based measures, rather than all unbounded information-theoretic measures.
3. The added constant in second-order Bayes factor utility is 2, rather than 1.

**Table 10.** Overview of preregistered measures of relevance. All notation is the same as Table 1, and *e* refers to Euler’s constant.

**Family**	**Measure Name**	**Order**	**Definition**
Direct	probability change	1st	|*p* − *q*|
	commitment change	2nd	|*c* − *d*|
	concentration change	2nd	|*α*_0_ + *β*_0_ − (*α*_1_ + *β*_1_)|
Entropy	entropy change	1st	|H(**q**) − H(**p**)|
	second-order entropy change	2nd	|H(B(*α*_1_, *β*_1_)) − H(B(*α*_0_, *β*_0_))|
KL divergence	KL utility	1st	*g*(KL(**q**‖**p**))
	second-order KL utility	2nd	*g*(KL(B(*α*_1_, *β*_1_)‖B(*α*_0_, *β*_0_)))
Bayes factor	Bayes factor utility	1st	1 − BF_*polar*_(*p*, *q*)^*a*^
	second-order Bayes factor utility	2nd	log(|*α*_1_ − *α*_0_| + |*β*_1_ − *β*_0_| + 2)

Further differences in how measures are computed in the preregistered study concern how free parameters were optimized. For each second-order measure, we use a linking function *κ* = *a* * *b*^*c*^ with two free parameters, *a* and *b*, to map a human commitment judgment *c* to concentrations of beta distributions *κ* (see Fitting Free Parameters section). In the preregistered study, for each second-order measure, we optimize these parameters jointly to maximize the Pearson correlation between the measure and the relevance judgments for the pilot data. This gives the values in Table 11. In the study presented in the main body of the paper, we jointly optimize *a*, *b*, and the parameter of the transformation function *r*. We also optimize them to instead minimize the mean square error, as we found that maximizing the Pearson correlation sometimes results in transformations that do not make full use of the range [0, 1), but rather map all values typically encountered in our data very close to 0.

**Table 11.** Parameters for the linking function *κ* = *a* * *b*^*c*^ following the preregistered protocol.

**Measure**	*a*	*b*
concentration change	1	1.97
second-order entropy change	1.76	0.98
second-order KL utility	4.83	1.79
second-order Bayes factor utility	2.44	3.81

### Results

#### Hyothesis 1: First-Order Belief Change Explains Relevance Rating.

The estimated posterior mean for the relevant slope coefficient is 2.948 (94.4% HDI: 2.317, 3.568), compared to the non-preregistered result 2.945 (94.4% HDI: 2.261, 3.601). The estimated posterior probability of the slope being positive is 1, compared to the non-preregistered result 1. The estimated log-probability difference for LOO model comparison is 2235.75 (standard error of estimate: 57.414), compared to the non-preregistered result 348.89 (standard error of estimate: 23.87). Both comparisons are significant under a simple *z*-score test. Our conclusions from the preregistered and non-preregistered results are the same.

#### Hypothesis 2: commitment change Additionally Contributes to Relevance Rating.

The estimated posterior mean for the relevant slope coefficient is 0.318 (94.4% HDI: [0.222, 0.421]), compared to the non-preregistered result 0.319 (94.4% HDI: [0.217, 0.417]). The estimated posterior probability of the slope being positive is 1, compared to the non-pregistered result 1. The better model under leave-one-out (LOO) model comparison is the model with the factor commitment change. The estimated log-probability difference for LOO model comparison is 53.513 (standard error of estimate: 11.143), compared to the non-pregistered result 53.071 (standard error of estimate: 11.137). Both comparisons are significant. Our conclusions from the preregistered and non-preregistered results are the same.

#### Hypothesis 3: Bayes Factor Utility Is the Best Predictor of Relevance Ratings.

Figure 14 shows the estimated expected log-likelihoods for LOO-CV, compared to the non-preregistered results in Figure 9. The best model uses Bayes factor utility as the single predictor of relevance ratings. When we directly compare Bayes factor utility to the second-best single-factor predictor, second-order Bayes factor utility, the estimated log-probability difference for LOO model comparison is 168.167 (standard error: 37.036), compared to the non-preregistered result 201.404 (standard error: 20.814). Both comparisons are significant. Our conclusions from the preregistered and non-preregistered results regarding this hypothesis are the same. The only notable difference is that second-order entropy change had the lowest expected log-likelihood following the preregistered protocol, but is fourth lowest following the non-preregistered protocol and has a substantially higher expected log-likelihood. This is likely due to the use of the scaling function *g* and better optimization of free parameters in the non-preregistered protocol.

## References

[bib1] Agha, O., & Warstadt, A. (2020). Non-resolving responses to polar questions: A revision to the QUD theory of relevance. Proceedings of Sinn und Bedeutung, 24(1), 17–34. 10.18148/sub/2020.v24i1.850

[bib2] Benz, A., Jäger, G., & van Rooij, R. (Eds.). (2006). Game theory and pragmatics. Palgrave Macmillan. 10.1057/9780230285897

[bib3] Blutner, R., & Zeevat, H. (Eds.). (2004). Optimality theory and pragmatics. Palgrave Macmillan. 10.1057/9780230501409

[bib4] Bürkner, P.-C., & Charpentier, E. (2020). Modelling monotonic effects of ordinal predictors in Bayesian regression models. British Journal of Mathematical and Statistical Psychology, 73(3), 420–451. 10.1111/bmsp.12195, 31943157

[bib5] Carcassi, F., & Franke, M. (2023). How to handle the truth: A model of politeness as strategic truth-stretching. In M. Goldwater, F. K. Anggoro, B. K. Hayes, & D. C. Ong (Eds.), Proceedings of the 45th Annual Conference of the Cognitive Science Society (pp. 222–228). Cognitive Science Society.

[bib6] Clark, H. H. (1979). Responding to indirect speech acts. Cognitive Psychology, 11(4), 430–477. 10.1016/0010-0285(79)90020-3

[bib7] Cover, T. M., & Thomas, J. A. (1991). Elements of information theory (1st ed.). Wiley. 10.1002/0471200611

[bib8] Crawford, V. P., & Sobel, J. (1982). Strategic information transmission. Econometrica, 50(6), 1431–1451. 10.2307/1913390

[bib9] Cummins, C., & Franke, M. (2021). Rational interpretation of numerical quantity in argumentative contexts. Frontiers in Communication, 6, 662027. 10.3389/fcomm.2021.662027

[bib10] Fagin, R., & Halpern, J. Y. (1994). Reasoning about knowledge and probability. Journal of the ACM, 41(2), 340–367. 10.1145/174652.174658

[bib11] Frank, M. C., & Goodman, N. D. (2012). Predicting pragmatic reasoning in language games. Science, 336(6084), 998. 10.1126/science.1218633, 22628647

[bib12] Franke, M. (2014). Typical use of quantifiers: A probabilistic speaker model. In P. Bello, M. Guarini, M. McShane, & B. Scassellati (Eds.), Proceedings of the 36th Annual Conference of the Cognitive Science Society (pp. 487–492). Cognitive Science Society.

[bib13] Franke, M., & Degen, J. (2023). The softmax function: Properties, motivation, and interpretation. PsyArXiv. 10.31234/osf.io/vsw47

[bib14] Gamut, L. (1990). Logic, language, and meaning, Volume 1: Introduction to logic. University of Chicago Press. 10.7208/chicago/9780226791678.001.0001

[bib15] Gazdar, G., & Good, D. (1982). On a notion of relevance. In N. Smith (Ed.), Mutual knowledge (pp. 88–100). Academic Press.

[bib16] Ginzburg, J. (1996). Dynamics and the semantics of dialogue. In J. Seligman & D. Westerståhl (Eds.), Logic, language, and computation (pp. 221–237). CSLI Publications.

[bib17] Good, I. J. (1950). Probability and the weighing of evidence. Griffin.

[bib18] Goodman, N. D., & Stuhlmüller, A. (2013). Knowledge and implicature: Modeling language understanding as social cognition. Topics in Cognitive Science, 5(1), 173–184. 10.1111/tops.12007, 23335578

[bib19] Grice, H. P. (1975). Logic and conversation. In P. Cole & J. L. Morgan (Eds.), Speech acts (pp. 41–58). Brill. 10.1163/9789004368811_003

[bib20] Hawkins, R., & Goodman, N. (2017). Why do you ask? The informational dynamics of questions and answers. PsyArXiv. 10.31234/osf.io/j2cp6

[bib21] Hawkins, R., Tsvilodub, P., Bergey, C. A., Goodman, N. D., & Franke, M. (2025). Relevant answers to polar questions. Philosophical Transactions of the Royal Society B: Biological Sciences, 380(1932), 20230505. 10.1098/rstb.2023.0505, 40808460 PMC12351304

[bib22] Herbstritt, M., & Franke, M. (2019). Complex probability expressions & higher-order uncertainty: Compositional semantics, probabilistic pragmatics & experimental data. Cognition, 186, 50–71. 10.1016/j.cognition.2018.11.013, 30743012

[bib23] Jeffrey, R. (1965). The logic of decision. University of Chicago Press.

[bib24] Jeffreys, H. (1961). Theory of probability (3rd ed.). Oxford University Press.

[bib25] Kao, J. T., Wu, J. Y., Bergen, L., & Goodman, N. D. (2014). Nonliteral understanding of number words. Proceedings of the National Academy of Sciences, 111(33), 12002–12007. 10.1073/pnas.1407479111, 25092304 PMC4143012

[bib26] Kass, R. E., & Raftery, A. E. (1995). Bayes factors. Journal of the American Statistical Association, 90(430), 773–795. 10.1080/01621459.1995.10476572

[bib27] Kubinec, R. (2023). Ordered beta regression: A parsimonious, well-fitting model for continuous data with lower and upper bounds. Political Analysis, 31(4), 519–536. 10.1017/pan.2022.20

[bib28] Kullback, S., & Leibler, R. A. (1951). On information and sufficiency. Annals of Mathematical Statistics, 22(1), 79–86. 10.1214/aoms/1177729694

[bib29] Lambert, B. (2018). A student’s guide to Bayesian statistics. Sage Publications. 10.4135/9781036234546

[bib30] Levinson, S. C. (1989). A review of relevance. Journal of Linguistics, 25(2), 455–472. 10.1017/S0022226700014183

[bib31] Lewis, D. (1969). Convention: A philosophical study. Harvard University Press. 10.1002/9780470693711

[bib32] Lin, J. (2016). On the Dirichlet distribution [Master’s thesis, Queen–s University]. Queen–s University Research Data Repository. https://mast.queensu.ca/∼communications/Papers/msc-jiayu-lin.pdf

[bib33] Merin, A. (1999). Information, relevance, and social decisionmaking: Some principles and results of decision-theoretic semantics. In L. S. Moss, J. Ginzburg, & M. de Rijke (Eds.), Logic, language, and computation (Vol. 2, pp. 179–221). CSLI Publications.

[bib34] Merrill, W., Warstadt, A., & Linzen, T. (2022). Entailment semantics can be extracted from an ideal language model. In A. Fokkens & V. Srikumar (Eds.), Proceedings of the 26th Conference on Computational Natural Language Learning (CoNLL) (pp. 176–193). Association for Computational Linguistics. 10.18653/v1/2022.conll-1.13

[bib35] Moss, S. (2015). On the semantics and pragmatics of epistemic vocabulary. Semantics and Pragmatics, 8, 5. 10.3765/sp.8.5

[bib36] Nowak, M. A., & Krakauer, D. C. (1999). The evolution of language. Proceedings of the National Academy of Sciences, 96(14), 8028–8033. 10.1073/pnas.96.14.8028, 10393942 PMC22182

[bib37] Özenoglu, Ö., Kallioinen, N., & Franke, M. (2023). faintr: Factor interpreter for Bayesian regression models [R package]. https://github.com/michael-franke/faintr

[bib38] Parikh, P. (1991). Communication and strategic inference. Linguistics and Philosophy, 14(5), 473–514. 10.1007/BF00632595

[bib39] Qing, C., Goodman, N. D., & Lassiter, D. (2016). A rational speech-act model of projective content. In A. Papafragou, D. Grodner, D. Mirman, & J. C. Trueswell (Eds.), Proceedings of the 38th Annual Conference of the Cognitive Science Society (pp. 1110–1115). Cognitive Science Society.

[bib40] Raiffa, H., & Schlaifer, R. (1961). Applied statistical decision theory. MIT Press.

[bib41] Roberts, C. (2012). Information structure in discourse: Towards an integrated formal theory of pragmatics. Semantics and Pragmatics, 5, 6. 10.3765/sp.5.6

[bib44] Rothe, A., Lake, B. M., & Gureckis, T. M. (2016). Asking and evaluating natural language questions. In A. Papafragou, D. Grodner, D. Mirman, & J. C. Trueswell (Eds.), Proceedings of the 38th Annual Conference of the Cognitive Science Society (pp. 2051–2056). Cognitive Science Society.

[bib45] Rothe, A., Lake, B. M., & Gureckis, T. M. (2018). Do people ask good questions? Computational Brain & Behavior, 1(1), 69–89. 10.1007/s42113-018-0005-5

[bib46] Rothe, A., Lake, B. M., & Gureckis, T. M. (2019). Asking goal-oriented questions and learning from answers. In A. K. Goel, C. M. Seifert, & C. Freksa (Eds.), Proceedings of the 41st Annual Conference of the Cognitive Science Society (pp. 981–986). Cognitive Science Society.

[bib47] Savage, L. J. (1951). The theory of statistical decision. Journal of the American Statistical Association, 46(253), 55–67. 10.1080/01621459.1951.10500768

[bib48] Scontras, G., Tessler, M. H., & Franke, M. (2017). Probabilistic language understanding: An introduction to the Rational Speech Act framework [Online course]. https://www.problang.org/

[bib49] Shannon, C. E. (1948). A mathematical theory of communication. Bell System Technical Journal, 27(3), 379–423. 10.1002/j.1538-7305.1948.tb01338.x

[bib50] Skyrms, B. (2010). Signals: Evolution, learning, and information. Oxford University Press. 10.1093/acprof:oso/9780199580828.001.0001

[bib51] Spence, A. M. (1973). Job market signaling. Quarterly Journal of Economics, 87(3), 355–374. 10.2307/1882010

[bib52] Sperber, D., & Wilson, D. (1987). Précis of *Relevance: Communication and cognition*. Behavioral and Brain Sciences, 10(4), 697–710. 10.1017/S0140525X00055345

[bib53] Sperber, D., & Wilson, D. (1995). Relevance: Communication and cognition (2nd ed.). Blackwell.

[bib54] Stalnaker, R. (1978). Assertion. In P. Cole (Ed.), Syntax and semantics, Volume 9: Pragmatics (pp. 315–332). Brill. 10.1163/9789004368873_013

[bib55] Stevens, J. S., Benz, A., Reuße, S., & Klabunde, R. (2016). Pragmatic question answering: A game-theoretic approach. Data & Knowledge Engineering, 106, 52–69. 10.1016/j.datak.2016.06.002

[bib56] Sumers, T. R., Ho, M. K., Griffiths, T. L., & Hawkins, R. D. (2024). Reconciling truthfulness and relevance as epistemic and decision-theoretic utility. Psychological Review, 131(1), 194–230. 10.1037/rev0000437, 37589706

[bib57] Tsvilodub, P., Franke, M., Hawkins, R., & Goodman, N. (2023). Overinformative question answering by humans and machines. In M. Goldwater, F. K. Anggoro, B. K. Hayes, & D. C. Ong (Eds.), Proceedings of the 45th Annual Conference of the Cognitive Science Society (pp. 1439–1445). Cognitive Science Society.

[bib58] Van Kuppevelt, J. (1995). Discourse structure, topicality and questioning. Journal of Linguistics, 31(1), 109–147. 10.1017/S002222670000058X

[bib42] van Rooij, R. (2004). Cooperative versus argumentative communication. Philosophia Scientiae, 8(2), 195–209. 10.4000/philosophiascientiae.576

[bib43] van Rooy, R. (2004). Utility, informativity and protocols. Journal of Philosophical Logic, 33(4), 389–419. 10.1023/B:LOGI.0000036830.62877.ee

[bib59] Vehtari, A., Gelman, A., & Gabry, J. (2017). Practical Bayesian model evaluation using leave-one-out cross-validation and WAIC. Statistical Computing, 27(5), 1413–1432. 10.1007/s11222-016-9696-4

[bib60] Wagner, E. O. (2015). Conventional semantic meaning in signalling games with conflicting interests. British Journal for the Philosophy of Science, 66(4), 751–773. 10.1093/bjps/axu006

[bib61] Wärneryd, K. (1993). Cheap talk, coordination, and evolutionary stability. Games and Economic Behavior, 5(4), 532–546. 10.1006/game.1993.1030

[bib62] Warstadt, A. (2022). Presupposition triggering reflects pragmatic reasoning about utterance utility. In M. Degano, T. Roberts, G. Sbardolini, & M. Schouwstra (Eds.), Proceedings of the 23rd Amsterdam Colloquium (pp. 444–451). ILLC, University of Amsterdam.

[bib63] Warstadt, A., & Agha, O. (2022). Testing Bayesian measures of relevance in discourse. Proceedings of Sinn und Bedeutung, 26, 865–886. 10.18148/sub/2022.v26i0.1034

[bib64] Wilson, D., & Sperber, D. (2004). Relevance theory. In L. R. Horn & G. Ward (Eds.), The handbook of pragmatics (pp. 607–632). Blackwell.

[bib65] Winterstein, G. (2012). What *but*-sentences argue for: An argumentative analysis of *but*. Lingua, 122(15), 1864–1885. 10.1016/j.lingua.2012.09.014

[bib66] Yoon, E. J., Tessler, M. H., Goodman, N. D., & Frank, M. C. (2020). Polite speech emerges from competing social goals. Open Mind: Discoveries in Cognitive Science , 4, 71–87. 10.1162/opmi_a_00035, 33225196 PMC7672308

